# Mechanisms of and mitigating strategies for cellular immune responses to CRISPR-associated nucleases in genome editing therapy

**DOI:** 10.3389/fmed.2026.1930231

**Published:** 2026-08-28

**Authors:** Bryan Hu, Venkata Sumanth Reddy Gutty, Roland W. Herzog, Dongsheng Duan

**Affiliations:** 1 Department of Molecular Microbiology and Immunology, School of Medicine, University of Missouri, Columbia, MO, United States; 2 Department of Pathobiology and Integrative Biomedical Sciences, College of Veterinary Medicine, University of Missouri, Columbia, MO, United States; 3 Department of Pediatrics, Herman B Wells Center for Pediatric Research, Indiana University, Indianapolis, IN, United States; 4 Department of Chemical and Biomedical Engineering, College of Engineering, University of Missouri, Columbia, MO, United States; 5 Department of Neurology, School of Medicine, University of Missouri, Columbia, MO, United States

**Keywords:** AAV, Cas9, clinical trial, CRISPR, CTL, genome editing, immune response, LNP

## Abstract

Immunogenicity of CRISPR-associated nucleases (Cas) is a critical barrier to the development of safe and effective genome editing therapies. These proteins inherently pose a risk of immune recognition due to their prokaryotic origins. A multitude of factors, such as the delivery vehicle, the route of administration, components of the therapeutics, tissue microenvironment, and pre-existing immunity, also contribute to the complexity of the host immune response to Cas proteins. As CRISPR-based therapies advance into clinical settings, it is imperative to elucidate and address the immunogenicity of Cas proteins. Here, using Cas9 as an example, we review the current understanding of Cas protein immunogenicity, the challenges it poses for therapeutic application, and strategies to mitigate cellular immune responses to Cas proteins.

## 1 Introduction

Clustered regularly interspaced short palindromic repeats (CRISPR) were first observed in 1987 by Yoshizumi Ishino and colleagues when sequencing DNA from *Escherichia coli* bacteria (1). These repetitive elements were later identified as a distinct genomic feature in prokaryotes by Francisco Mojica in the early 2000s, who also inferred their role in prokaryotic immunity (2, 3). In 2007, studies by Barrangou and Horvath provided experimental evidence that CRISPR functions as an adaptive immune system in prokaryotes (4). In 2012, CRISPR, along with CRISPR-associated nucleases (Cas), was adapted for programmable genome editing by Jennifer Doudna and Emmanuelle Charpentier (5), rapidly transforming the field of gene editing. Since its invention, many CRISPR-based technologies have emerged, expanding beyond conventional nuclease-mediated genome editing to include RNA targeting and precision editing approaches such as base and prime editing (6, 7). Although the full clinical potential of these technologies remains to be realized, significant progress has already been made toward therapeutic development. In 2020, the first *in vivo* CRISPR-Cas9 gene editing therapy, EDIT-101, was administered to a patient with a rare condition called Leber’s congenital amaurosis 10 (LCA10) in a clinical trial (NCT03872479) (8). In 2021, Gillmore et al. provided the first demonstration of the safety and efficacy of intravenously delivered CRISPR-Cas9 therapy for another rare hereditary disease, transthyretin amyloidosis (ATTR) (NCT04601051) (9). A major milestone occurred in 2023, when exagamglogene autotemcel (CASGEVY) became the first CRISPR-Cas9 gene editing therapy to receive FDA approval for the treatment of sickle cell disease (SCD) and transfusion-dependent beta-thalassemia (TDT) (10, 11).

Given Cas proteins’ bacterial origin, their immunogenicity has been perceived as a critical factor for therapeutic success. Of these, Cas9 is used the most. In this review, using Cas9 as an example, we summarize current understanding of immune responses to Cas proteins and discuss emerging strategies to mitigate their immunogenicity.

## 2 Cas9 as an immunological antigen

### 2.1 Bacterial origin of Cas9

CRISPR-Cas9 is a programmable gene-editing tool adapted from the microbial adaptive immune system. CRISPR, together with Cas proteins, provides sequence-specific protection against invading bacteriophages (5, 12). Two of the most prominent Cas9 orthologues are derived from *Streptococcus pyogenes* (*S. pyogenes*; SpCas9) and *Staphylococcus aureus* (*S. aureus*; SaCas9). Both *S. pyogenes* and *S. aureus* are bacterial pathogens that frequently colonize humans (13). In fact, pre-existing humoral and cellular adaptive immunity have been detected against both SpCas9 and SaCas9 in healthy individuals, reflecting a high prevalence of natural exposure to these two bacteria (14, 15). Upon subsequent exposure to Cas9, individuals with pre-existing immunity may mount a humoral response against the protein. Cas9-specific antibodies can bind and neutralize the protein, forming immune complexes and marking it for rapid clearance. In parallel, professional antigen-presenting cells (APC) may internalize extracellular Cas9 protein and process it into antigenic peptide fragments within endolysosomal compartments. These peptides can be loaded onto major histocompatibility complex (MHC) II molecules and be presented to CD4+ T cells. Activation of CD4+ T cells can provide helper functions that boost antibody production and contribute to cellular immune responses toward Cas9. In addition, endogenously synthesized Cas9 can be processed by the proteasome and presented on MHC I molecules to CD8+ T cells. Interaction between Cas9-derived peptide-MHC (pMHC) complexes with cognate T cell receptors, along with appropriate co-stimulatory signals, can initiate proliferation and effector differentiation, ultimately leading to immune-mediated cellular clearance of Cas9-expressing cells.

### 2.2 Overview of Cas9 structure and function

CRISPR-Cas9 genome editing relies on the Cas9 nuclease and the formation of a ribonucleoprotein (RNP) complex with a guide RNA (gRNA) to generate targeted double-stranded breaks (DSBs) at specific genomic loci containing a compatible protospacer adjacent motif (PAM).

The Cas9 protein consists of two major lobes: the recognition (REC) lobe and the nuclease (NUC) lobe (16). The REC lobe comprises the REC1, REC2, and REC3 domains, which collectively recognize the gRNA and mediate conformational changes required for efficient cleavage of target DNA (17). The NUC lobe contains the PAM-interacting (PI) domain, histidine-asparagine-histidine (HNH) nuclease domain, and the repair of UV-damaged DNA endonuclease component C (RuvC) nuclease domain. The PI domain facilitates recognition and binding to the PAM, triggering DNA strand separation and enabling gRNA/DNA heteroduplex formation (16). Upon binding to the target DNA, the HNH domain cleaves the DNA strand complementary to the gRNA, while the RuvC domain cleaves the non-complementary strand, generating a DSB (18).

Following the induction of DSBs, the cell initiates DNA damage repair (DDR) pathways to fix the damaged DNA. Classically, there are two major DDR pathways: non-homologous DNA end joining (NHEJ) and homology-directed repair (HDR). NHEJ is the predominant repair pathway in mammalian cells for fixing double-strand breaks across different phases of the cell cycle (19). This pathway depends on the Ku protein to stabilize DNA ends and on a DNA ligase complex to rejoin them (19). This process can lead to small insertions or deletions (indels) of nucleotides at the break site, which potentially may introduce frameshifts or other disruptive mutations (20). In contrast, HDR is primarily active during the S and G2 phases of the cell cycle (21). Furthermore, this process requires a homologous DNA template to repair the DNA damage and generally introduces fewer sequence errors (22).

### 2.3 Cas9 structure and immunodominant epitopes

Ferdosi et al. identified immunodominant human CD8+ T-cell epitopes in SpCas9 (23). Using an epitope prediction model that prioritizes MHC binding affinity and residue hydrophobicity, the authors ranked candidate epitopes for human MHC class I allele HLA-A*02:01. Among the top 5 predicted epitopes, two 9-mer epitopes labeled α (NLIALSLGL) and β (ILEDIVLTL) were classified as immunodominant. Both epitopes are localized to the REC lobe, with peptide α residing in the REC2 domain and peptide β in the REC3 domain. They also identified two subdominant epitopes: the 10-mer peptide γ (YLNAVVGTAL) and the 9-mer peptide δ (GLFGNLIAL). Peptide γ is mapped to the RuvC domain of the NUC lobe, while peptide δ is in the REC2 domain. The fifth unnamed peptide (ILADANLDKV) was located within the PI domain. Immunodominant human CD8+ T cell epitopes for HLA-A*02:01 have also been mapped for SaCas9 and AsCas12a (24, 25). Three immunodominant epitopes in SaCas9 are located at the RuvC-I domain (GLDIGITSV) and the PI domain (VTVKNLDVI and ILGNLYEVK) (24, 25). Three immunodominant epitopes in AsCas12a are located at the REC domain (RLITAVPSL and LNEVLNLAI) and the RuvC-II domain (YLSQVIHEI) (24).

Simhadri et al. identified SaCas9 peptides presented on MHC class II molecules in human dendritic cells (DC) using mass spectrometry (26). They employed the MHC-associated peptide proteomics (MAPPs) assay, in which pMHC II complexes were affinity-purified from mature DCs cultured with SaCas9. Purified pMHC II complexes were subsequently analyzed through liquid chromatography-tandem mass spectrometry (LC-MS/MS) to identify peptide fragments. Of the 681 peptides initially discovered through MAPPs, 22 distinct peptides were identified to be associated with increased CD4+ T cell proliferation. Nine peptides were localized to the REC lobe, while the remaining 13 peptide fragments mapped throughout the NUC lobe.

## 3 Antigen processing and cellular immune activation

Adaptive immune responses are mediated by T lymphocytes, which eliminate infected cells or assist B cells in providing protection against extracellular pathogens. However, T cells cannot directly sample pathogenic threats. Instead, antigen presentation systems have evolved to display peptide fragments derived from synthesized or internalized proteins on MHC molecules. Engagement between the pMHC and its cognate T cell receptor (TCR) initiates T cell activation. Full T cell activation additionally requires a second co-stimulatory signal, most commonly CD28, to avoid T cell anergy (27–29). A third signal, provided by the local cytokine milieu, further shapes T cell differentiation and functional outcomes (30). Naïve T cells primarily recirculate secondary lymphoid organs and do not directly survey peripheral tissues (21). As such, tissue-resident or migratory APCs, particularly DCs, acquire antigens in peripheral tissues and subsequently migrate to lymph nodes, where they activate naïve T cells. Once activated, T cells can execute specialized effector functions that lead to the cytotoxic elimination of target cells or to the coordination of cellular immune responses through helper activity.

### 3.1 MHC class I presentation of Cas9

**FIGURE 1:**
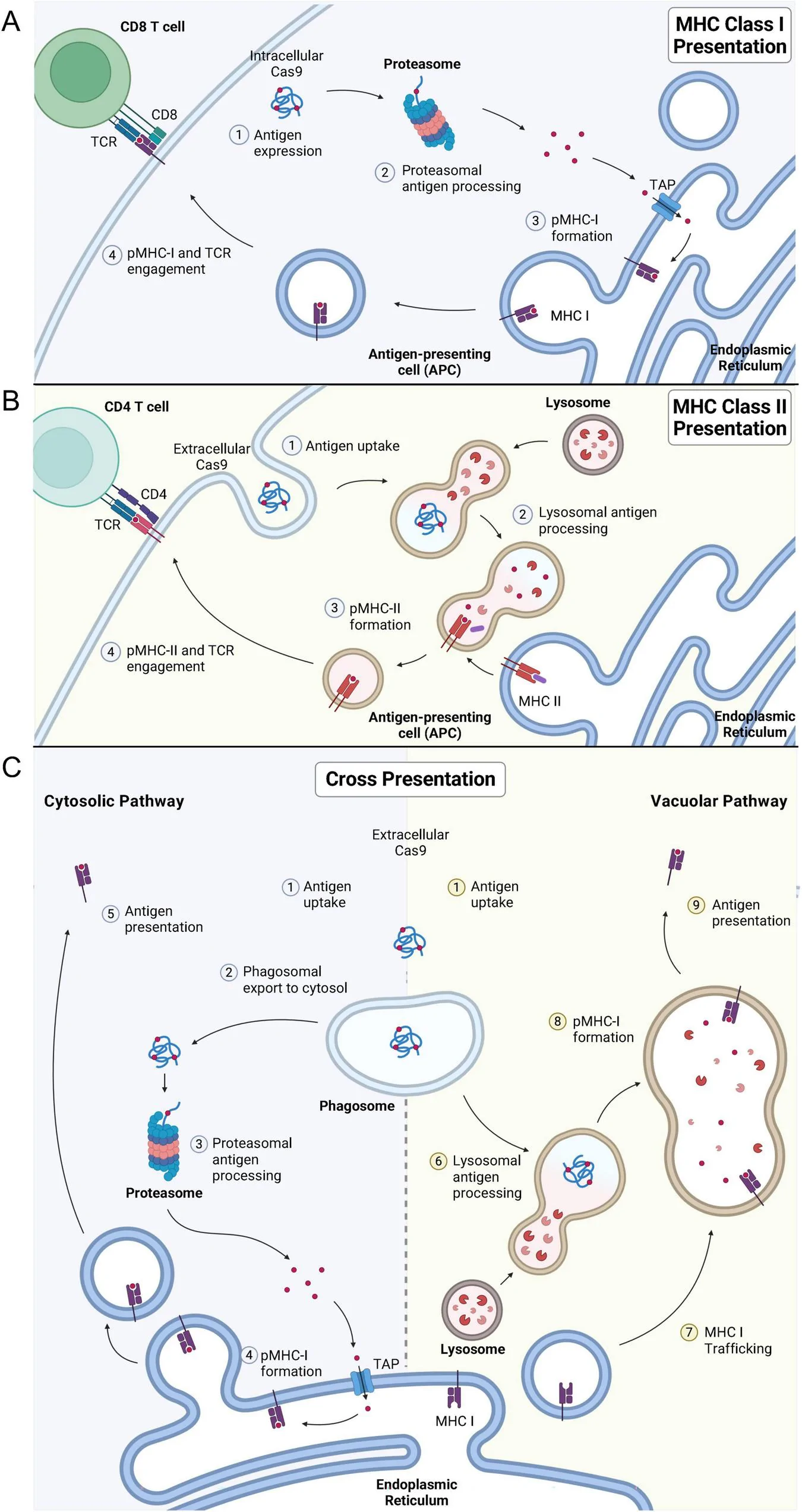
Antigen processing and presentation pathways for Cas9. **(A)** MHC class I presentation. Following antigen expression (1), cytosolic Cas9 is processed by the proteasome (2). Cas9-derived peptides are transported into the ER via TAP and loaded onto MHC class I molecules (3). The peptide-MHC I complex is transported to the cell surface for recognition by CD8+ T cells (4). **(B)** MHC class II presentation. Extracellular Cas9, either delivered as an RNP complex or released from dying neighborhood Cas9-expressing cells, is internalized by antigen-presenting cells (1). Endocytosed Cas9 is processed in the endolysosomal compartment (2). MHC class II molecules traffic to the endolysosomal compartment, where invariant chain degradation permits loading of Cas9-derived peptides (3). The peptide-MHC II complex is subsequently transported to the cell surface for recognition by CD4+ T cells (4). **(C)** Cross presentation. Antigen-presenting cells may route exogenous Cas9 through either a cytosolic pathway or a vacuolar pathway. In the cytosolic pathway, Cas9 is taken up (1) and exported to the cytosol (2) for proteasomal processing (3). The resulting peptides are transported into the ER via the TAP for loading onto MHC class I molecules (4). The peptide-MHC I complex is subsequently transported to the cell surface for CD8+ T cell recognition (5). In the vacuolar pathway, Cas9 is taken up (1) and the Cas9-containing phagosome fuses with the lysosome. Cas9 is processed within the resulting endolysosomal compartment (6). MHC class I molecules traffic to the endolysosomal compartment (7). Cas9-derived peptides are loaded onto MHC class I molecules (8) and the resulting peptide-MHC I complex is transported to the cell surface for CD8+ T cell recognition (9). ER, endoplasmic reticulum; pMHC, peptide-MHC complex; RNP, ribonucleoprotein; TAP, transporter associated with antigen processing; TCR, T cell receptor.

The Cas9 protein and its orthologs contain immunostimulatory epitopes that can activate adaptive immune cells (14, 23–26). Following intracellular expression, Cas9 is degraded into short peptide fragments by proteasomal complexes (Figure 1A). These peptides are transported into the endoplasmic reticulum by the transporter associated with antigen processing (TAP) (31). A subset of these antigenic peptides is processed and loaded on MHC class I molecules, forming the pMHC I complex (32). This complex is subsequently trafficked from the endoplasmic reticulum to the cell surface, where it awaits recognition by TCRs (33). Effector CD8+ T cells that recognize the epitope presented on Cas9-expressing cells initiate cytotoxic clearance through perforin- and granzyme B-mediated killing, as well as Fas-Fas ligand interactions (34). In a canine Duchenne muscular dystrophy (DMD) model, CD8+ granzyme B+ T cells were observed surrounding degenerating muscle fibers following CRISPR-based therapy, suggesting T cell-mediated cytotoxicity (35).

Major histocompatibility complex class I immunostimulatory epitopes vary in amino acid sequence and can range in length from 8 to 15 residues depending on the MHC alleles, but most are approximately 9 amino acids in length (36). Although many epitopes may be generated from Cas9, not all peptides elicit a strong adaptive immune response. One or a few epitopes may dominate T cell responses, a phenomenon known as immunodominance (37). Consistent with this, Ferdosi et al. evaluated *in silico*-predicted SpCas9 MHC class I epitope peptides using peripheral blood mononuclear cells (PBMCs) from HLA-A*02:01 donors in an IFN-gamma (IFN-γ) ELISpot assay (23). 83% of donors exhibited positive immunoreactivity against at least one Cas9-derived peptide, of which five distinct peptides were identified to be immunodominant amongst donors.

### 3.2 MHC class II presentation of Cas9

Major histocompatibility complex class II molecules are constitutively expressed by professional APCs, including DCs, macrophages, and B cells (38). These APCs can sample Cas9 antigens via various internalization pathways, including endocytosis, phagocytosis, and macropinocytosis. Following uptake, Cas9 is processed in endolysosomal compartments to generate short antigenic peptide fragments of 13–25 residues in length (39) (Figure 1B). Concurrently, MHC class II molecules are synthesized in the endoplasmic reticulum with an invariant chain glycoprotein (Ii, CD74) bound to its peptide binding groove, which prevents premature peptide loading (40). The MHC class II complex is trafficked to endolysosomal compartments, where it binds a high-affinity peptide following invariant chain degradation, forming a mature pMHC II complex (33). The pMHC II complex is subsequently transported to the cell surface for recognition by CD4+ T cells. Engagement of the CD4+ TCR with pMHC II initiates T cell activation. Upon activation, CD4+ T cells undergo clonal expansion, differentiate into specialized functional subsets, secrete cytokines, and activate B cells and macrophages, thereby contributing to the adaptive immune response of Cas9.

The functional outcome of CD4+ T cell activation depends heavily on the nature and activation state of professional APCs, as well as the surrounding cytokine milieu. For example, CD8α^+^ DCs are associated with T-helper 1 (Th1) differentiation, whereas CD8α^–^ DCs are involved in T-helper 2 (Th2) differentiation (41). Upon TCR engagement and with appropriate costimulatory signaling, CD4+ T cells differentiate into specialized effector subsets in response to specific cytokine signals (42). Th1 differentiation is triggered by interleukin-12 (IL-12) and IFN-γ and is characterized by the transcription factor T-bet, promoting immunity against intracellular pathogens. In contrast, Th2 differentiation is driven by IL-4 signaling and GATA-binding protein 3 (GATA3), mounting immune responses against extracellular parasites. T-helper 17 (Th17) cells arise from transforming growth factor-beta (TGF-beta), IL-6, and IL-23 signaling, and their differentiation is primarily mediated by RAR-related orphan receptor gamma t (RORγt), in response to extracellular bacterial and fungal threats. Regulatory T cells (Tregs) differentiation is triggered by TGF-beta and IL-2 signaling and driven by forkhead box protein 3 (FOXP3). Tregs suppress immune responses and induce tolerance (42). T follicular helper (Tfh) cells are induced by IL-6 and IL-21, characterized by Bcl-6 expression, and mediate humoral immunity through interaction with B cells (43).

Each CD4+ subset maintains distinct effector functions that collectively contribute to the immune response. In the context of CRISPR exposure, these differentiation pathways may direct immune responses toward cytotoxic clearance of edited cells or establishment of immune tolerance. In support of this, Hakim et al. reported co-recruitment of CD4+ T cells and CD8+ T cells in CRISPR-treated muscle, accompanied by elevated proinflammatory muscle cytokines such as IL-2, which promote the survival and expansion of cytotoxic T-lymphocytes (CTL) (35).

### 3.3 Cross presentation of Cas9

Cross-presentation is a unique process in which exogenous antigens are presented in a pMHC I complex to activate CD8+ cytotoxic T cells (44) (Figure 1C). Although the precise cellular biochemical mechanisms are not well defined, it is thought that cross-presentation follows two principal pathways: the vacuolar pathway and the endosome-to-cytosol pathway (45) (Figure 1C). In the cytosol pathway, internalized antigens such as Cas9 are translocated from the endosome or phagosome to the cytosol (46, 47). There, antigenic peptides are generated following ubiquitylation and proteasomal degradation. These peptides are then trafficked to the endoplasmic reticulum and are loaded onto MHC I molecules to generate mature pMHC I complexes. In the vacuolar pathway, internalized antigens are processed and proteolytically digested to generate peptides. MHC I molecules are recruited to endolysosomes, allowing antigenic peptides to be loaded. This process is proteasome-independent and can be upregulated by toll-like receptor signaling (48).

## 4 Innate immunity

Innate immune recognition of CRISPR components can reduce therapeutic efficacy and prime adaptive immune responses. These immune responses are mediated by pattern recognition receptors (PRRs), which detect foreign nucleic acids and proteins in a manner contingent on the delivery modality and immune context of CRISPR therapy. For example, in the context of RNP delivery, retinoic acid-inducible gene I (RIG-I) can recognize 5′-triphosphate in *in vitro*-transcribed gRNAs, leading to the induction of type I interferon responses (49). Notably, RIG-I-mediated immune responses can be reduced or eliminated by phosphatase-mediated removal of 5′-triphosphate or with chemically synthesized gRNAs (49). Similarly, Toll-like receptor 9 (TLR9) can sense unmethylated CpG motifs in the adeno-associated virus (AAV) genome in AAV-mediated CRISPR therapy. Upon recognition of unmethylated CpGs, TLR9 upregulates pro-inflammatory cytokine production, thereby promoting the adaptive immune response (50–54).

In addition to nucleic acid sensing, innate immune responses can also be directed against the Cas9 protein itself. This may occur through exogenous Cas9 (e.g., Cas9 in RNP or released from dying cells). Kang et al. demonstrated that exogenous Cas9 exposure to human PBMCs activated the stimulator of interferon genes (STING)-signal transducer and activator of transcription 6 (STAT6) signaling pathway (55). The upstream sensor responsible for this has yet to be elucidated, as knockout of cyclic GMP-AMP synthase (cGAS), the canonical activator of STING, failed to block pathway activation.

Additionally, the active editing process itself creates genomic DSBs and micronuclei, which may activate the cGAS-STING pathway (56). This is thought to subsequently recruit TANK-binding kinase 1 (TBK1) and activate interferon regulatory factor 3 (IRF3) through phosphorylation (57). In parallel, STING signaling can engage the inhibitor of κB kinase (IKK), promoting activation of the nuclear factor of kappa light-chain enhancer of activated B cells (NF-κB) (58). Collectively, these signaling pathways coordinate the production of type I interferons and pro-inflammatory cytokines, which can prime and amplify adaptive immune responses.

## 5 Pre-existing cellular immunity to Cas9

Pre-existing adaptive immunity to Cas9 poses a translational constraint that threatens the efficacy of CRISPR gene therapy. Antigen-specific T cell immunity is commonly assessed through antigen recall assays using PBMCs or lymphoid tissue-derived lymphocytes, with T-cell activation determined by pro-inflammatory cytokine secretion using enzyme-linked immunospot (ELISpot), activation-induced surface markers (flow cytometry), clonal expansion (proliferation assays), or direct antigen recognition (MHC multimer/tetramer staining). Pre-existing Cas9-specific T-cell immunity has been detected in human cohorts following stimulation with the full-length Cas9 protein or peptides. Charlesworth et al. detected SaCas9 and SpCas9-specific T cell reactivity in 14 (78%) and 12 (67%) of 18 donors, respectively (14). Wagner et al. stimulated PBMCs from 48 donors with SpCas9 and found that 96% of donors responded with effector T cell activation, evidenced by upregulation of the activation-induced marker CD137 (15). Similarly, using the IFN-γ ELISpot assay, Ferdosi et al. detected the SpCas9 peptide-specific T cell response in 83% of 12 donors (23).

## 6 Treatment-induced immune responses to Cas9

CRISPR-based gene editing can be achieved either *ex vivo* or *in vivo*. Both approaches have the potential to elicit host immune responses depending on various factors such as delivery modality, pre-existing immunity, and treatment approach. As immune responses to Cas9 have been identified as a critical consideration for therapeutic safety and efficacy, it is essential to evaluate the immunological and clinical outcomes observed in past and ongoing CRISPR studies.

CASGEVY (exagamglogene autotemcel; exa-cel) is the first FDA-approved *ex vivo* CRISPR-Cas9 gene editing treatment for SCD and TDT. It entails electroporation of an RNP complex composed of Cas9 and a sgRNA targeting a GATA-1 binding motif within the erythroid-specific enhancer region of the *BCL11A* gene in autologous CD34+ hematopoietic stem and progenitor cells (HSPCs) (10, 11). The *BCL11A* gene encodes a transcription factor that represses the genes encoding the γ-globin chains of fetal hemoglobin (HbF) (59, 60). Upon disruption of the enhancer, BCL11A expression is reduced, de-repressing γ-globin and increasing HbF production during erythroid differentiation. The edited HSPCs are intravenously infused back into the patient following myeloablation. Because Cas9 is absent from the final cell product, patients are not directly exposed to Cas9, theoretically circumventing Cas9-directed immune responses. The safety and efficacy of this approach were evaluated in two pivotal phase 3 trials in patients with SCD (CLIMB SCD-121) and TDT (CLIMB THAL-111) (10, 11). Across both trials, 97% of patients with SCD were free from severe vaso-occlusive crises, and 91% of patients with TDT achieved transfusion independence for at least 12 months. The overall safety profile was comparable to that of a traditional autologous HSPC transplantation, with all serious adverse events resolved, and no graft failure or malignancy observed. Importantly, no Cas9-specific immune reactions were reported, suggesting that Cas9 immunogenicity does not pose a barrier to therapeutic efficacy in the *ex vivo* autologous setting.

EDIT-101 represents the first *in vivo* CRISPR-Cas9 gene-editing therapy evaluated in human patients. It leverages an AAV5 vector to deliver SaCas9 from the photoreceptor-specific GRK1 promoter, and two highly specific sgRNAs targeting intron 26 of the *CEP290* (IVS26 variant) gene (8). Approximately 77% of patients with Leber’s congenital amaurosis type 10 (LCA10) harbor an intronic point mutation in intron 26 of the *CEP290* gene. The mutation introduces a cryptic splice site, resulting in the inclusion of an aberrant exon and disruption of CEP290 protein expression (8, 61). EDIT-101 removes the point mutation in intron 26 to prevent aberrant splicing. The safety and efficacy of this approach were evaluated in the BRILLIANCE phase 1–2 clinical trial (NCT03872479), which enrolled 12 adult and 2 pediatric patients with LCA10. Patients received a single subretinal injection of EDIT-101 across four dose-escalating cohorts. Adult patients received 6 × 10^11^ vg/mL (low dose), 1 × 10^12^ vg/mL (mid-dose), or 3 × 10^12^ vg/mL (high dose). Pediatric patients received the mid-dose of 1 × 10^12^ vg/mL. Results were promising but therapeutic efficacy was modest and heterogeneous. Meaningful improvements were observed in at least one key visual outcome in 9 of 14 patients (65%). However, only 4 of 14 (29%) demonstrated clinically meaningful improvement in best-corrected visual acuity. No serious adverse events or dose-limiting toxic effects were observed, although 22 ocular adverse events were recorded across participants. Pre-existing immunity to SaCas9 was detected in 5 (36%) of 14 patients. Intriguingly, immunity to SaCas9 disappeared in 3 patients following treatment. No SaCas9 antibodies were detected before or after the treatment, suggesting that humoral immune responses were not induced by subretinal delivery of EDIT-101. The immune-privileged subretinal space, restricted photoreceptor-specific expression, and perioperative immunosuppression may collectively contribute to the limited Cas9-directed immune response.

In contrast to the AAV-based EDIT-101, NTLA-2001 (nexiguran ziclumeran; nex-z) and NTLA-2002 use lipid nanoparticle (LNP) to deliver the CRISPR editing machinery. NTLA-2001 is designed to treat ATTR, ATTR with cardiomyopathy (ATTR-CM), and ATTR with polyneuropathy (ATTRv-PN) (9, 62–65). NTLA-2002 is designed to treat hereditary angioedema (9, 62–65). Both therapeutics utilized LNP to deliver SpCas9 mRNA and a sgRNA (targeting the *TTR* gene and the *KLKB1* gene in NTLA-2001 and NTLA-2002, respectively). Phase 1–2 studies of NTLA-2001 and NTLA-2002 demonstrated robust, dose-dependent reductions in circulating levels of the target protein. In the phase 2 trial, NTLA-2002 reduced total plasma kallikrein of up to 55% at 25 mg and 86% at 50 mg by week 16. Similarly, a single infusion of NTLA-2001 achieved a mean of 90% reduction in serum transthyretin at 12 months across all dose cohorts. Both therapies were generally well tolerated, with no dose-limiting toxicities reported. One severe adverse event in the NTLA-2001 ATTR-CM phase 1 trial was attributed to an infusion-related reaction rather than direct therapy toxicity. Transient elevations in alanine aminotransferase (ALT) and aspartate aminotransferase (AST) were reported across multiple NTLA-2001 and NTLA-2002 cohorts. These AST and ALT elevations resolved within 8 weeks of onset without medical intervention. Whether these transient transaminase elevations reflect T cell-mediated cellular immune responses to Cas9 protein expressed in hepatocytes remains to be established. With respect to humoral immunity, anti-Cas9 antibodies were detected in all 10 patients enrolled in the NTLA-2002 phase 1 trial and in 21 of 36 patients (58%) in the NTLA-2001 ATTRv-PN phase 1 trial. In neither trial did anti-Cas9 antibody production appear to interfere with pharmacokinetics, pharmacodynamics, or safety outcomes. Following encouraging phase 1–2 results for NTLA-2001 and NTLA-2002, phase 3 trials MAGNITUDE (ATTR-CM, NCT06128629), MAGNITUDE-2 (ATTRv-PN, NCT06672237), and HAELO (hereditary angioedema) were initiated. However, safety concerns emerged in the MAGNITUDE trial. An 80-years-old participant receiving NTLA-2001 was hospitalized following detection of grade 4 liver transaminase elevation and increased total bilirubin (66). The patient had multiple comorbidities and died shortly after hospitalization (67). This event differs from the transient grade 1–2 liver transaminase elevations reported from phase 1–2 studies (62, 65), raising the possibility that severe hepatic immune-mediated injury may occur in a subset of patients. The underlying cause, including the potential role of cellular anti-Cas9 immunity, remains under investigation.

Clinical trial NCT06594094, sponsored by HuidaGene Therapeutics, used HG302, a CRISPR-based therapeutic that aims to restore dystrophin expression via exon skipping or reframing (68). HG302 is a single “all-in-one” AAV9 vector that expresses hfCas12Max, an engineered high-fidelity Cas12 variant (69), and a sgRNA targeting the human DMD gene exon 51 splicing donor site. In humanized ΔEx52 DMD mice, HG302 restored dystrophin expression in more than 60% of myofibers. HG302 was also assessed monthly for 3 months in healthy 1-year-old cynomolgus macaques at two doses (5 × 10^13^ vg/kg and 2 × 10^14^ vg/kg). Antigen-specific T-cell reactivity toward hfCas12Max and AAV9 was assessed by IFN-γ ELISpot assays in PBMCs. T-cell reactivity to hfCas12Max was reported to be minimal in the low-dose group. However, the data from the high-dose NHP was not reported. Importantly, cynomolgus macaques are not naturally colonized by Lachnospiraceae bacterium, a source bacterium of Cas12, and may not properly recapitulate pre-existing human anti-Cas12 immunity. As such, preclinical NHP immunogenicity data should be interpreted with caution as they may underestimate immune responses encountered in human patients. There was no evidence of hepatorenal toxicity. Neutralizing antibodies to AAV9 were detected (1:1,250∼1:6,250), along with anti-drug antibodies against AAV9 (1:1,600∼1:6,400) and hfCas12Max (1:200∼1:800). A clinical trial (NCT06594094) was initiated. Two patients (6 and 8.4-years-old) received low-dose HG302 (low 10^13^ vg/kg). Muscle biopsy at day 90 from the first patient (8.4-years-old) detected the AAV vector genome, but the hfCas12Max transcript was barely detectable, and there was minimal on-target editing and dystrophin restoration. No severe adverse events were observed in the first two patients, but the 4th patient died from acute respiratory distress syndrome (ARDS) within days after receiving a higher dose of HG302 (70). Immunological data from human patients remains to be disclosed (68).

Another clinical trial (NCT06392724) used the cytosine base-editing therapeutic GEN6050X to induce exon skipping in DMD patients (71). This strategy employs two AAV9 vectors. One delivers the base editor, and the other delivers gRNA. A preclinical study in a humanized mouse model showed significant dystrophin restoration. In cynomolgus macaques, some liver toxicity was observed at 13 weeks at the doses of 5 × 10^13^ vg/kg and 2 × 10^14^ vg/kg, but was fully resolved by week 26. Three ambulatory patients (6.5–10 years of age) were treated at a dose of 5 × 10^13^ vg/kg while receiving immunosuppression with rituximab and sirolimus. Anti-Cas9 antibody levels increased rapidly, starting at 1-week post-injection. ELISpot revealed minimal Cas9-specific T cell response at 6 months. The first patient was diagnosed with ARDS at day 5 and subsequently recovered. The western blot from the second patient showed a 2.4-fold increase in dystrophin expression relative to baseline.

A nonambulatory DMD patient died 8 days following an *in vivo* CRISPR editing therapy (72, 73). This therapy used an AAV9 vector encoding dCas9 fused to the VP64 transactivation domain (dSaCas9-VP64), along with a sgRNA targeting the cortical dystrophin isoform Dp427c, to upregulate transcription. The patient received an intravenous dose of 1 × 10^14^ vg/kg of the therapeutic vector. Despite having undetectable pre-existing anti-AAV9 antibody titers, undergoing prophylactic immune suppression (rituximab, glucocorticoids, and sirolimus), and screening negative for infectious diseases, the patient developed a severe innate immune response. This response led to a cytokine-mediated capillary leak syndrome, and the patient eventually succumbed to pericardial effusion and ARDS. Post-mortem studies revealed findings consistent with an AAV-specific response. Vector genome biodistribution showed high vector genome copy numbers in the liver (680 vg per diploid genome), as expected, but unexpectedly high copies in the lung (119 vg per diploid genome). Copy numbers in the skeletal muscle were comparable to those in the lung but were lower in myocardium (34–48 vg per diploid genome). dSaCas9 transcript and protein were undetectable except for trace amounts in the liver. IFN-γ ELISpot assays using the patient’s PBMC at day 4 or 7 revealed no transgene-specific T-cell responses. AAV9 neutralizing antibodies remained negative throughout the study. Of note, ARDS occurred across all three CRISPR-based DMD clinical trials, with fatal outcomes reported in this trial and the HuidaGene Therapeutics-sponsored trial, potentially reflecting an AAV-associated innate immune response. Taken together, these cases highlight the importance of delivery modality and its impact on both therapeutic efficacy and safety, particularly in patients with pre-existing inflammatory conditions.

Collectively, these early clinical studies not only demonstrate encouraging efficacy but also offer some insights into the immunological safety of CRISPR-Cas9 in humans. As expected, *ex vivo* CRISPR editing appears to pose nominal Cas9-specific immune risks (10, 11). In contrast, the immunological safety of Cas9 in *in vivo* CRISPR editing therapies remains less well characterized, and several limitations confound interpretation of available data. First, the clinical studies discussed here enrolled small patient cohorts. In addition to limited statistical power, small patient study sizes underrepresent diverse HLA backgrounds and patient populations. Second, all studies employed prophylactic immunosuppression regimens, which may actively suppress Cas9-directed immune responses. Accordingly, the absence of an immune response in immunosuppressed patients should not be interpreted as evidence of inherent immunological safety. Third, most clinical studies discussed here fail to interrogate or report T-cell responses to Cas9. The absence of T-cell data should not be interpreted as a negative T-cell response. Furthermore, even when T-cell assays are performed, PBMC-based ELISpot approaches cannot sample localized populations (tissue-resident or infiltrating CTLs) where clinically relevant cytotoxicity occurs. Clearly, efficacy and safety are governed not only by the therapeutic itself, but also by host-specific and context-dependent variables, underscoring the need for further investigation into the determinants of Cas9 immunogenicity and therapeutic outcome.

## 7 Determinants of cellular immunogenicity

Numerous factors may influence cellular immunogenicity toward Cas9, many of which are interconnected and collectively shape the overall immune response. Among these, antigen persistence, antigen load, delivery modality, route of administration, host HLA restriction, and tissue context and microenvironment are key determinants of Cas9 immunogenicity in CRISPR therapy.

### 7.1 Antigen persistence and load

Antigen persistence refers to the duration an antigen remains in the host and is available for immune recognition. The persistence of Cas9 antigen can greatly contribute to host immune responses, as prolonged antigen availability can sustain antigen presentation and increase T cell activation. Several variables can influence antigen persistence, including the delivery platform, the duration and magnitude of protein expression, and the intrinsic stability of the protein. Furthermore, the lifespan of protein-expressing cells, which is influenced by cellular turnover or immune-mediated clearance, may also determine antigen persistence.

The delivery modality of Cas9 can greatly impact antigen persistence in CRISPR-Cas9 therapy. For example, delivery of Cas9 as an RNP results in minimal antigen persistence because it is rapidly degraded following delivery. Similarly, Cas9 mRNA delivery results in short antigen persistence after Cas9 mRNA degradation. In contrast, viral vectors such as AAV can persist as episomes, leading to long-lasting expression and prolonged antigen presentation. Vector design elements that influence transcriptional activity, such as promoter strength and mRNA stability, may further shape the duration of Cas9 expression, thereby increasing antigen persistence. In addition to vector-related factors, the intrinsic stability of Cas9 contributes to antigen persistence and antigen availability for presentation.

The concept of antigen load refers to the total quantity and diversity of antigens encountered by the host immune system. Several factors contribute to antigen load, including the initial dose administered and the magnitude and duration of antigen expression. Antigen load may shape the host immune response at several levels. In the context of CD8+ T-cell activation, a higher antigen load may recruit a larger pool of antigen-specific CD8^+^ T cells and drive their expansion into effector populations, thereby amplifying cellular immune responses (74, 75). However, over time, responses initiated at lower antigen loads may “catch up” to those induced by higher antigen loads, suggesting that elevated antigen levels primarily influence the early recruitment of CD8+ T cells (75–77). Evidence from vaccination studies suggests that a greater antigen load results in a more robust immune response, but the resulting effector CD8+ T cells also exhibit lower functional avidity (78). The loss of functional avidity was attributed to clonal deletion, tolerance induction, and terminal differentiation and exhaustion of T cells (78). Conversely, lower doses may promote higher functional avidity and contribute to a greater response. Thus, antigen load influences the kinetics of the immune response and the balance between immunity and tolerance (79, 80). The balance between response magnitude and functional quality, and the propensity for differentiation and exhaustion ultimately shape the overall immune outcome (81–83).

### 7.2 Delivery modality and route of administration

The current landscape of CRISPR therapy utilizes the Cas9 endonuclease delivered as DNA, RNA, or protein. Expression cassettes can be delivered effectively using viral DNA vectors such as AAV, lentiviral, and adenoviral vectors. Both lentiviral and AAV delivery greatly increase the risk of an anti-Cas9 adaptive immune response due to greater antigen exposure from lentiviral integration and AAV episomal persistence. Additionally, lentiviral vectors raise concerns about genotoxicity due to insertional mutagenesis, while AAV is limited by its small packaging capacity (84, 85). While adenoviruses can accommodate a larger payload, they also elicit potent innate and adaptive responses that may destroy the transduced cells (86, 87). Nonviral methods, such as LNP, virus-like particles, and extracellular vesicles, offer the benefit of transient expression. LNPs may allow repeat administration and scale to systemic doses. However, infusion responses, complement, and anti-polyethylene glycol responses require careful consideration. Virus-like particles and extracellular vesicles remain constrained by manufacturing and characterization (88, 89). *Ex vivo*-edited cell products minimize Cas9 exposure and enable rigorous quality control. However, this approach has limitations in the range of treatable tissues and the need for extensive patient conditioning.

Route of administration is a major determinant of the immune response to Cas9 in CRISPR therapy, as it affects biodistribution, and thereby immune surveillance exposure. Systemic intravenous delivery allows for widespread biodistribution of the therapeutic payload. However, it increases exposure to circulating APCs and resident APCs in the liver, spleen, and lymphoid tissues, thereby increasing the risk of both humoral and cell-mediated immunity. Additionally, systemic delivery may trigger complement activation and is limited by pre-existing immunity against viral vectors. In contrast, localized delivery reduces widespread antigen exposure. However, the immune outcome of localized delivery depends heavily on the target tissue. For example, intramuscular injections may induce local tissue injury and inflammation. Furthermore, muscle-resident APCs can take up antigens and initiate adaptive immune responses. On the other hand, localized delivery to immune-privileged tissues such as the eye via intraocular delivery may elicit a dampened or tolerated immune response to the antigen. The interaction between the route of administration and the subsequent immune response toward Cas9 remains a clinically important but poorly investigated question.

### 7.3 Tissue context and microenvironment

The tissue microenvironment in which Cas9 is expressed can strongly influence the host immune response. Certain organs are considered immune-privileged organs, including the eye and brain, where immune surveillance and inflammatory responses are tightly regulated (90–92). Similarly, there are immune-tolerant organs (such as the liver) that may favor a regulatory or anti-inflammatory immune response (79, 93, 94). In contrast, certain organs (such as skin and lung) promote a strong pro-inflammatory response (95, 96). Furthermore, inflammatory microenvironments present in certain diseases (such as muscular dystrophy) may also enhance antigen presentation and immune activation.

#### 7.3.1 Immune privileged organs

Immune privilege is a property whereby immune responses are stringently regulated to limit collateral tissue damage, particularly in tissues with limited regenerative capacity, such as the central nervous system (CNS). The eye is a classic and extensively documented site of immune privilege, whose privilege status is conferred by three principal characteristics: the blood-retinal barrier (BRB), an inhibitory microenvironment, and active regulation of immune responses (90, 97). The BRB is a highly specialized microvascular barrier composed of retinal vascular endothelial cells, pericytes, and glial cells that create tight junctions to maintain ocular homeostasis and restrict the entry of circulating immune cells, potential toxins, and pathogens (98, 99). The ocular inhibitory microenvironment comprises both soluble and membrane-bound immunosuppressive factors (100). Soluble factors such as TGF-beta, α-melanocyte-stimulating hormone, and vasoactive intestinal peptide contribute to ocular immune privilege by promoting anti-inflammatory cytokine production and induction of Tregs (97). Membrane-bound inhibitory ligands such as programmed death-ligand 1 (PD-L1), Fas ligand, thrombospondin-1, and galectins protect the eye by engaging infiltrating immune cells and inducing anergy, apoptosis, or Treg differentiation (98, 100). Active regulation of immune responses is exemplified by anterior chamber-associated immune deviation (ACAID). In ACAID, antigens introduced into the anterior chamber are processed by resident F4/80^+^CD11b^+^ ocular APCs that migrate to the thymus and the spleen to preferentially activate regulatory T cells (101, 102). This mechanism promotes systemic antigen-specific tolerance and suppresses both Th1- and Th2-mediated immunity. However, this immune privilege may be limited in its ability to prevent inflammation under high antigen load. For example, intravitreal AAV administration has been shown to elicit clinically significant ocular inflammation, particularly at higher vector doses (103, 104).

Beyond the eye, immune privilege is a feature of other anatomical sites, most notably the CNS. Like the eye, the CNS is protected by the blood-brain barrier, a structure analogous to the BRB, composed of tight junctions among cerebrovascular endothelial cells, pericytes, and astrocytes that restrict the passage of immune cells and large molecules (105). The CNS parenchyma lacks classical lymphatic drainage, although it is supported by meningeal lymphatic and glymphatic clearance systems (106). It maintains an immunoregulatory microenvironment with anti-inflammatory cytokine secretion, including TGF-beta, IL-10, and IL-4, as well as neuron-microglia inhibitory signaling pathways such as CD200-CD200R, CX3CL1-CX3CR1, and CD47–SIRPα. Together, these signals regulate microglial activity and maintain microglial homeostasis, preserving a surveillant and non-inflammatory state (105). Altogether, these immune-privileged features make the eye and CNS attractive targets for Cas9 gene therapy, as local immunoregulation may mitigate pre-existing Cas9 immunity. However, whether these sites are truly protected from vector and Cas9-driven inflammatory complications remains unclear and warrants further investigation.

#### 7.3.2 Immune tolerant organs

The liver is a unique organ with an immunosuppressive microenvironment, enabling it to induce local and systemic tolerance to self and foreign antigens (93, 94). The immunosuppressive microenvironment is largely driven by specialized resident cells that express anti-inflammatory mediators and inhibitory cell-surface ligands, such as IL-10, TGF-beta, and PD-L1 (107, 108). These resident cells include resident DCs, plasmacytoid DCs, liver sinusoidal endothelial cells, Kupffer cells, and hepatic stellate cells (109). These specialized resident cells shape the liver’s uniquely tolerogenic microenvironment by promoting anti-inflammatory pathways either directly or indirectly through processes such as clonal deletion, clonal anergy, clonal deviation, T-cell exhaustion, and Treg induction (109). In the context of Cas9 gene therapy, these tolerogenic mechanisms may limit the development of robust cellular immune responses against the Cas9 nuclease and its delivery vehicle. There is substantial evidence suggesting liver-directed AAV gene therapy may induce antigen-specific Tregs that modulate immune tolerance toward the transgene (110–117). The tolerogenic properties of the liver in the context of AAV-mediated transgene transfer are best exemplified in hemophilia gene therapy. Liver-targeted expression of human coagulation factor IX (F.IX) consistently elicits F.IX-specific CD4+ CD25+ FOXP3+ Tregs that confer durable immune tolerance that suppresses effector T cell responses (107, 118). Similar induction of tolerance has been demonstrated across a range of antigens, including the highly immunogenic model antigen ovalbumin (110). Whether Cas9, a large bacterial protein with broad pre-existing immunity, can be similarly tolerized by hepatic delivery remains an important open question. It should be noted that, depending on antigen doses and other factors, the liver is also capable of priming T cells *in situ* in an IL-1 receptor-dependent manner by formation of tertiary lymphoid structures (119).

#### 7.3.3 Proinflammatory microenvironments

Unlike immune-privileged or tolerant organs that favor anti-inflammatory responses, certain tissues and their microenvironments may be inherently pro-inflammatory, a tendency that can be further amplified by disease. The skeletal muscle microenvironment is generally viewed as immunologically active due to the presence of tissue-resident APCs capable of presenting antigens and recruiting other immune cells (120, 121). Similarly, the lung microenvironment is inherently pro-inflammatory, populated by specialized immune cells such as alveolar macrophages and airway epithelial cells that continuously survey for harmful agents and mount rapid, cytokine- and chemokine-mediated recruitment of immune cells (95, 122, 123). Certain genetic diseases, such as DMD, limb-girdle muscular dystrophy, cystic fibrosis, Pompe disease, and sickle cell disease, may further induce a chronically pro-inflammatory phenotype due to ongoing tissue damage, dysfunctional immune signaling, and failure to resolve inflammation. In DMD, loss of dystrophin drives cycles of myofiber necrosis that trigger a chronic inflammatory response characterized by infiltration of immune cells, including neutrophils, M1 macrophages, mast cells, and eosinophils (124). This is accompanied by a marked upregulation of pro-inflammatory cytokines, including TNF-alpha, IL-6, IFN-γ, and IL-17, and extensive chemokine-mediated immune recruitment that fails to resolve due to impaired M1 to M2 macrophage transition (124, 125). Under such pro-inflammatory conditions, immune responses to antigenic stimuli such as Cas9 may be substantially enhanced.

## 8 Strategies to mitigate Cas9 immunogenicity in CRISPR-based therapies

The immune response to Cas9 gene therapy is determined by a multitude of factors influencing antigen exposure, innate immune activation, and adaptive immune recognition. These include the magnitude and persistence of Cas9 expression, antigen processing and presentation, immunogenic properties, the tissue specificity of the vector, target tissue microenvironment, and the route of administration. Strategies aimed at limiting antigen burden, attenuating innate immune sensing, or suppressing adaptive immune activation hold promise for reducing Cas9 immunogenicity.

### 8.1 Protein engineering

**FIGURE 2:**
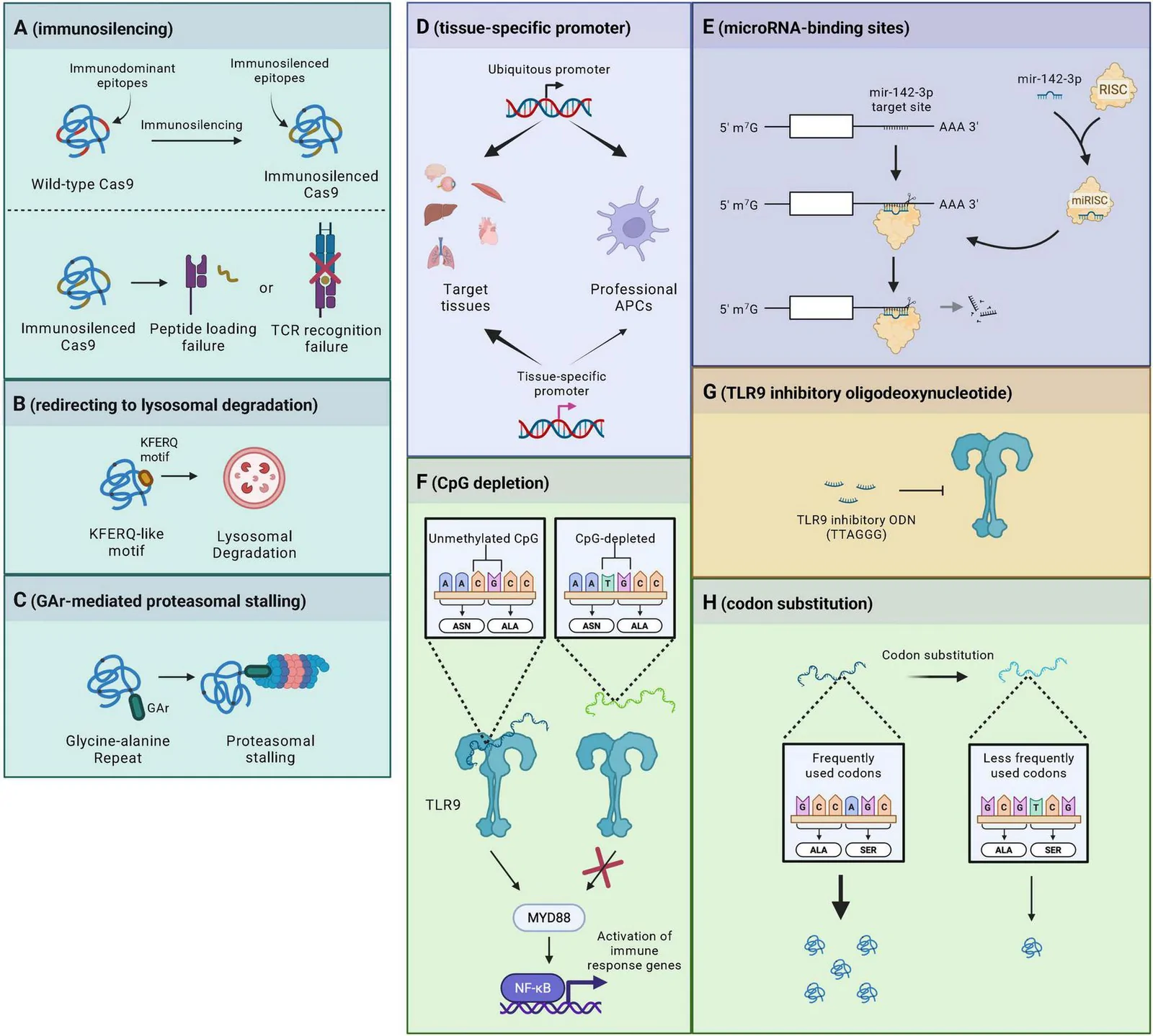
Strategies to reduce Cas9 immunogenicity through protein and nucleic acid engineering. Protein engineering strategies are shown in panels **(A–C)**. Nucleic acid engineering strategies are shown in panels **(D–H)**. **(A)** Immunosilencing. In this strategy, antigenic epitopes are immunosilenced by mutagenesis. The modified peptide either fails to bind MHC and form a stable peptide-MHC complex or forms a complex that cannot be recognized by the T cell receptor (TCR). **(B)** Redirecting cytosolic Cas9 for lysosomal degradation. In this strategy, a KFERQ-like motif is introduced to Cas9. This motif directs Cas9 to the lysosome for degradation. By avoiding proteasomal degradation, this strategy prevents peptide-MHC I complex formation and antigen presentation. **(C)** Glycine-alanine repeats (GAr)-mediated proteasomal stalling. In this strategy, GAr is fused to the N-terminal end of the Cas9 protein to prevent proteasomal degradation via stalling. **(D)** Tissue-specific promoter. In this strategy, Cas9 expression in professional antigen-presenting cells (APCs) is limited by the tissue-specific promoter. **(E)** MicroRNA binding site. In this strategy, the mir-142-3p target site is incorporated in the expression cassette. This leads to the selective degradation of the Cas9 mRNA in APCs. **(F)** CpG depletion. In this strategy, unmethylated CpGs are eliminated in the Cas9-encoding DNA to prevent TLR9 activation. **(G)** TLR9 inhibitory oligodeoxynucleotide (ODN). In this strategy, the TLR9 inhibitory ODN is incorporated in the expression cassette to suppress TLR9 activation. **(H)** Codon substitution. In this strategy, Cas9 expression (antigen load) is reduced by substituting the frequently used codons with non-optimal codons.

Cytotoxic T cell responses responsible for the clearance of Cas9-expressing cells arise from recognition of antigenic peptide fragments derived from the Cas9 nuclease. Engineering the Cas9 protein to reduce its immunogenicity, therefore, represents an attractive mitigation strategy. One approach involves generating immunosilenced Cas9 variants (Figure 2A). It centers on identifying potential antigenic epitopes that may be presented by pMHC molecules, using either computational prediction or experimental mapping. Given the large size of the Cas9 protein, many peptide fragments can theoretically be loaded onto MHC molecules. However, only a subset of these antigenic epitopes drives most of the T cell immune responses; these are known as immunodominant epitopes. Once identified, these immunodominant epitopes can be selectively mutated to prevent pMHC loading and/or TCR recognition. The proof of concept for this strategy was first demonstrated by Ferdosi et al. (23). The authors engineered an immunosilenced SpCas9 with reduced human CD8+ T cell immunogenicity. More recently, Zhang and colleagues extended this approach to additional Cas9 orthologues and generated immunosilenced variants of SaCas9 and AsCas12a (24). Immunosilenced SaCas9 variants were validated *in vivo* and displayed comparable on-target editing while exhibiting reduced T cell response compared to wild-type SaCas9 (24). It should be noted that targeted mutation of the Cas9 protein requires careful consideration of its impact on the structure and function of the nuclease. Novel immunosilenced Cas9 variants should be subject to functional validation of on-target editing before use.

A second strategy seeks to reduce Cas9 immunogenicity by redirecting intracellular degradation away from the proteasomal pathway. Proteasomal degradation is the primary source of peptide fragments for MHC Class I presentation (Figure 1A). Diverting Cas9 toward an alternative degradation route may result in a curtailed cytotoxic T cell response toward Cas9. This approach has been used to generate a fast-degrading SpCas9 variant (fdCas9) (126, 127). This variant harbors a KFERQ-like motif that directs the protein to lysosomes for selective degradation through chaperone-mediated autophagy (128–130) (Figure 2B). It is estimated that 40% of proteins in mammalian proteomes contain this motif (131). Incorporating the KFERQ-like motif into the exogenous Cas9 protein allows exploitation of endogenous mechanisms to direct Cas9 away from the proteasomal degradation pathway and toward the lysosomal degradation pathway. By preventing proteasomal degradation, Cas9 variants containing KFERQ-like motifs will not be loaded onto MHC I molecules (Figure 1A), reducing the chances of antigen presentation and CD8+ T cell activation. However, this strategy has important limitations. Professional APCs can sample fdCas9 either through direct transduction or exogenous Cas9 released from dying transduced cells. In such cases, lysosomal targeting could still result in MHC I presentation via cross presentation (Figure 1C). Moreover, redirection of fdCas9 within APCs may promote MHC II antigen presentation to CD4+ T cells, potentially amplifying the overall anti-Cas9 immune response. The effectiveness of this approach still lies in the proof-of-concept phase and requires rigorous validation before clinical translation.

A third approach draws on the immune evasion strategy of Epstein-Barr virus (EBV). The EBV nuclear antigen-1 (EBNA-1) encodes a glycine-alanine repeat (GAr) domain that enables the virus to remain undetected in host cells by limiting its own antigen presentation (132). Fusion of the GAr domain to Cas9 offers a potentially double-layered approach to reduce peptide display on MHC molecules while maintaining lower steady-state Cas9 protein levels. First, the GAr domain acts as a cis-acting inhibitor of the ubiquitin-proteasome pathway (133). Specifically, it is thought to interfere with the ATPase ring of the proteasome, causing proteasomal degradation to stall (134, 135) (Figure 2C). As such, fusion of the GAr domain to Cas9 may reduce immunogenicity by preventing proteasomal degradation, thereby limiting the pool of peptides available for MHC I presentation and loading. Second, the GAr domain may suppress translation of its own encoding mRNA (136, 137). By simultaneously reducing proteasomal processing and attenuating protein synthesis, the GAr domain provides a compelling framework for the rational engineering of Cas9 variants with diminished immunogenic potential. In support, GAr fusion has been used to attenuate the immunogenicity of tetracycline/doxycycline-inducible system for gene therapy applications (138, 139).

Modification of the Cas9 protein sequence may introduce novel immunogenic epitopes that are not present in the native sequence. For example, fusion proteins such as GAr-Cas9 may generate novel junctional peptides at the fusion site, while point mutations introduced to silence immunodominant epitopes may create new MHC-binding peptides for a different HLA allele. As such, neoepitope generation represents a distinct immunogenic liability of protein engineering strategies. All engineered Cas9 variants should be comprehensively evaluated for their immunogenicity and neoepitope potential prior to clinical translation.

### 8.2 Nucleic acid engineering

Payload modification presents unique approaches to mitigate both Cas9-specific and vector-related immune responses. One obvious approach is to limit Cas9 expression using a tissue-specific promoter (Figure 2D). Strong ubiquitous promoters will drive Cas9 expression broadly across all cell types, including professional APCs, thereby increasing both antigen load and exposure. In contrast, tissue-specific promoters confine Cas9 expression to target tissues, reducing off-target Cas9 expression while minimizing antigen presentation to T cells. However, tissue-specific promoters may not be uniformly stringent. Some tissue-specific promoter may still drive expression in APCs, which may be sufficient to sustain low-level antigen presentation.

Incorporation of microRNA-binding sequences is another powerful approach to restrict Cas9 expression in APCs. miR-142-3p is a microRNA closely associated with immune homeostasis and is highly expressed in hematopoietic and antigen-presenting cells (140, 141). Insertion of a miR-142-3p binding sites into the 3′ untranslated region of the Cas9 transgene will enable selective destabilization or translational repression of Cas9 transcripts in APCs (Figure 2E). This will minimize antigen presentation to T cells while preserving expression in the intended target tissues (142–145).

CpG depletion represents a well-characterized approach to reduce vector genome immunogenicity (Figure 2F). Unmethylated CpG motifs can be sensed by TLR9, leading to the production of type I interferon and subsequent activation of cytotoxic T lymphocytes (51, 52, 54, 146, 147). Consequently, CpG depletion from the transgene expression cassette may minimize vector genome-induced CTL responses and prolong transgene expression (148, 149). However, complete CpG elimination may be challenging in the context of AAV vector engineering, as vector yield is reduced when CpGs are depleted from the inverted terminal repeats, the essential viral components of the AAV vector (150). It should be pointed out that synonymous codon substitution required for CpG depletion may result in codon combinations that alter the translational efficiency of Cas9 due to codon usage bias. This may increase or reduce Cas9 expression depending on the availability of cognate tRNA (see discussion below). A complementary strategy to CpG depletion is the co-delivery of TLR9 inhibitory oligodeoxynucleotide (ODN) sequences within the vector genome (Figure 2G). The ODN repeat sequence TTAGGG, commonly found in mammalian telomeres, has been shown to inhibit TLR9 dimerization and downstream signaling (151–153). Inclusion of the TLR9 inhibitory ODN sequence may compensate for residual CpG contents that cannot be fully removed (154–156).

A further, largely unexplored strategy is to leverage codon usage bias to attenuate Cas9 expression and reduce overall antigen load (Figure 2H). Translational efficiency is partly determined by the availability of cognate tRNAs, which differ across species, tissue/cell types, and developmental or physiological states (157, 158). Because codon choice influences translational efficiency, synonymous codon substitutions can be used not only to optimize expression in a target tissue, but also to attenuate expression in unintended cell types, including APCs (159). By introducing non-optimal codons, Cas9 expression can be selectively reduced in tissues with low tRNA abundance for those codons, thereby limiting the Cas9 peptide pool for MHC presentation. However, this approach may be practically challenging, as codon usage bias is highly context-dependent, tissue-specific tRNA data is limited, and the relationship between codon optimization and protein folding is not yet well understood.

### 8.3 Transient expression of Cas9

**FIGURE 3:**
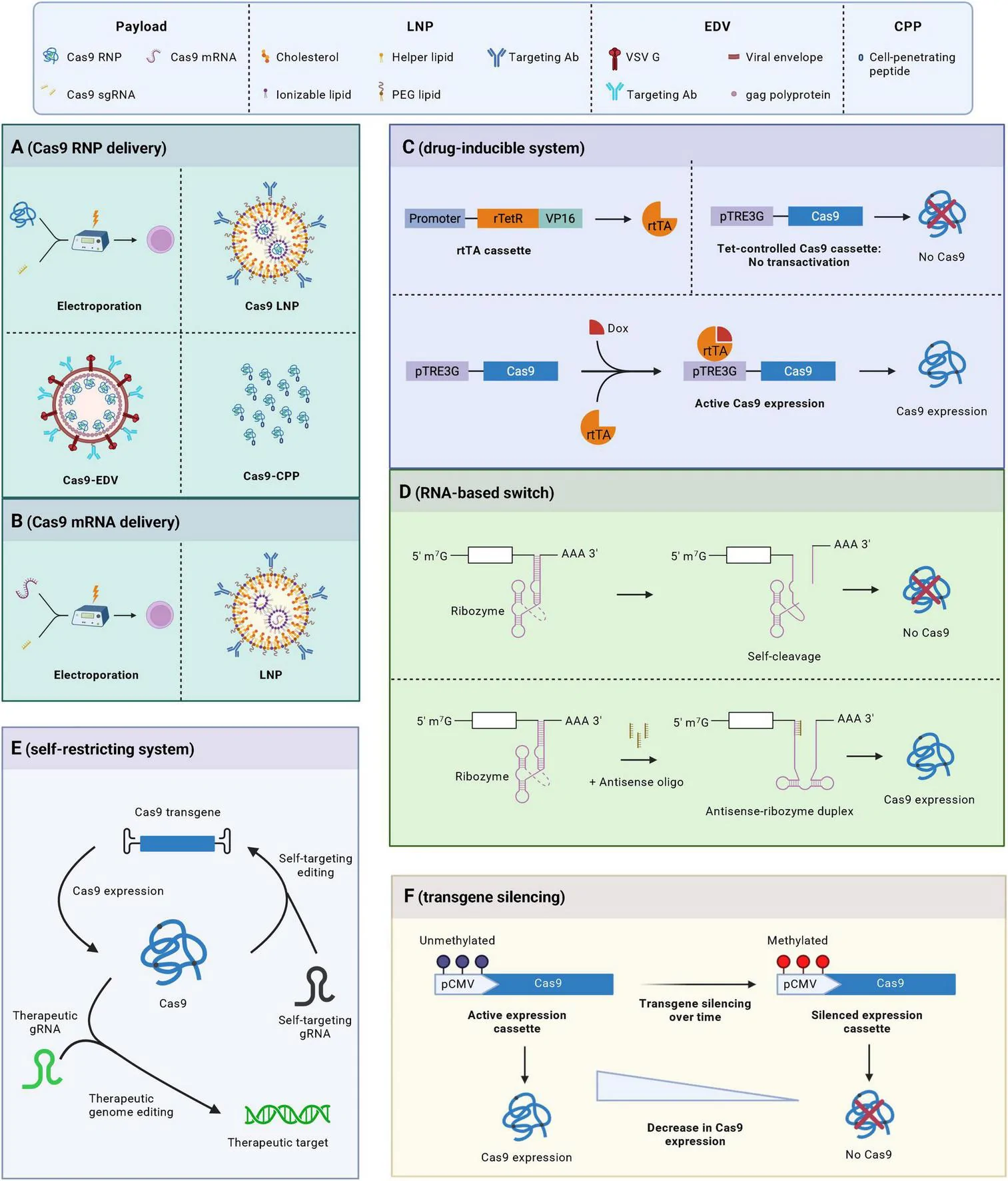
Approaches to limit Cas9 antigen persistence through transient expression. **(A)** Cas9 ribonucleoprotein (RNP) delivery. Cas9 RNP can be delivered *ex vivo* via electroporation. Cas9 RNP can also be delivered *in vivo* via lipid nanoparticle (LNP), enveloped delivery vehicle (EDV), or fusion with a cell-penetrating peptide (CPP). **(B)** Cas9 mRNA delivery. Cas9 mRNA can be delivered *ex vivo* via electroporation and *in vivo* via LNP. **(C)** Drug-inducible system. Cas9 expression can be controlled by a doxycycline (Dox)-inducible system where delivery of doxycycline allows rtTA to bind to pTRE3G and drive Cas9 expression. **(D)** RNA-based switch. In the absence of the antisense oligo, the riboswitch self-cleavage removes pA and leads to mRNA degradation. Addition of the antisense oligo alters the riboswitch structure and abolishes self-cleavage and mRNA degradation. **(E)** Self-inactivating System. This system incorporates an autoregulatory circuitry by including a self-targeting gRNA that prevents Cas9 expression after successful editing of the Cas9 expression cassette. **(F)** Transgene silencing. This strategy limits Cas9 expression through epigenetic silencing of the promoter and/or Cas9 coding-sequence.

The duration of antigen presentation contributes to the adaptive immune response (160–163). Transient expression of Cas9 is a potential strategy to circumvent this issue. The transient expression of Cas9 can be achieved in several ways. These include the direct delivery of Cas9 as a protein or mRNA, delivery of Cas9 genomes under the control of an inducible system, incorporation of self-restricting systems for CRISPR-Cas9, or regulation of Cas9 expression with transgene silencing (Figure 3).

#### 8.3.1 RNP and mRNA delivery of Cas9

Direct delivery of Cas9 protein to target tissues is expected to result in the shortest duration of antigen exposure among current delivery strategies (Figure 3A), as there is no renewable source for continued protein production. Consequently, the quantity of Cas9 present in the host is finite and transient. This delivery approach is particularly well-suited for *ex vivo* autologous gene editing, as exemplified by CASGEVY, in which Cas9 RNP and gRNA are electroporated into CD34+ HSPCs (10, 11) (Figure 3A).

Several Cas9 RNP platforms have been reported for *in vivo* delivery, including LNPs, enveloped delivery vehicles (EDVs), and cell-penetrating peptides (CPPs) (Figure 3A). LNP-mediated delivery of Cas9 RNP has achieved some success in the cornea, lung, and liver (164–167). EDVs are generated by fusing Cas9 to the HIV gag polyprotein. Although structurally similar to virus-like particles (VLPs), EDVs are engineered to exclude capsid-core-related proteins, thereby yielding a simplified and efficacious delivery system (168). EDV Cas9 RNP has resulted in robust *in vivo* editing in human T cells (169, 170). CPP-conjugated Cas9 RNP has also demonstrated success in extrahepatic tissues, such as the airway and skeletal muscle (171, 172). Despite the progress, efficient delivery of Cas9 protein to the target tissue remains technically challenging and may limit its applicability across therapeutic contexts. Furthermore, determining optimal dosing to achieve efficient editing while balancing an acceptable safety profile remains an important consideration.

Similarly, delivery of Cas9 mRNA is another means of achieving transient expression (Figure 3B), as protein expression ceases after mRNA degradation and protein turnover. Cas9 mRNA can be delivered via electroporation in the setting of *ex vivo* genome editing. Advances in LNP delivery systems have enabled efficient *in vivo* delivery of Cas9 mRNA to peripheral organs, particularly the liver, following systemic administration (9, 62–65). However, mRNA-based approaches face similar limitations in targeting less accessible tissues, which may reduce their applicability across various therapeutic contexts. Together, Cas9 protein and mRNA delivery offer a promising approach to achieve transient Cas9 expression to mitigate immunogenic risk. However, their delivery efficiency must be carefully considered in the context of specific diseases and therapeutic requirements.

#### 8.3.2 Inducible systems of Cas9

Since the discovery of the lac operon in bacteria, inducible expression systems have been widely researched, leading to the development of tetracycline-inducible systems for mammalian gene regulation. These regulatory platforms have been adapted to CRISPR-Cas9 applications primarily to reduce off-target genome editing and limit genotoxicity. Beyond these established applications, inducible control of Cas9 expression may also represent a viable strategy to circumvent Cas9 immunogenicity by limiting the duration and magnitude of antigen expression (173, 174).

Transcriptional control of Cas9 can be achieved using an inducible expression system such as the tetracycline response element 3rd generation (pTRE3G), which contains tet operator sequences, coupled with the expression of a reverse tetracycline-controlled transactivator (rtTA) (175–177) (Figure 3C). In the absence of doxycycline, rtTA does not bind to pTRE3G, and because the promoter lacks any endogenous mammalian transcription factor binding sites, it exhibits virtually no basal activity. Upon doxycycline introduction, rtTA undergoes a conformational change that enables it to bind the tet operator sequences, rendering the promoter transcriptionally active and allowing Cas9 to be expressed (178). Despite the appeal of the inducible system, several challenges warrant considerations. First, tetracycline-inducible systems are prone to leaky basal expression even under repressive conditions, which may be sufficient to sustain low-level antigen presentation (179–181). Second, the size of rtTA expression cassette may impose a packaging constraint in the context of AAV gene therapy, especially considering the large SpCas9 transgene. Third, the requirement to deliver rtTA as an additional component carries its own immunogenic liability, as rtTA is of bacterial origin. Finally, the pharmacokinetics of doxycycline dosing must be carefully tested to achieve consistent induction. Many of these issues can be partially addressed through a combination of strategies, such as splitting Cas9 across dual vectors, employing improved promoters and rtTA for greater repression and induction, and incorporating additional control such as the GAr domain, destabilizing domains or small-molecule-regulated Cas9 inhibitors to enforce tighter suppression of basal expression (138, 182–184).

Apart from drug-inducible systems, ribozyme-based RNA switches represent a novel approach to controlling Cas9 expression (185, 186) (Figure 3D). These systems offer two practical advantages for CRISPR gene therapy. First, RNA-based switches are generally under 200 bp, making them compact. Second, these switches do not require co-delivery of an extraneous regulatory protein, circumventing related immunogenic liabilities (187). To control Cas9 expression, RNA-based switches can be incorporated in the transgene expression cassette between the coding sequence of Cas9 and the polyA as a 3′ untranslated component. Under basal conditions, RNA-based switches contain a ribozyme that undergoes self-cleavage, removing the polyA tail from the mRNA transcript. When a complementary antisense oligonucleotide is provided, it binds to the ribozyme sequence, sterically blocking self-cleavage and stabilizing the transcript, thereby allowing Cas9 to be expressed. While most RNA-based switches suffer from a narrow regulatory range, some of these systems have been developed to achieve stringent leak repression and robust inducible expression (188). The primary limitation of ribozyme-based inducible systems lies in construct specificity. Different Cas9 orthologs and more complex architectures, such as intein-mediated or RNA-based split-Cas9, may each require distinct switch designs that need to be optimized and tested. Furthermore, the cost of synthesizing and delivering sufficient antisense oligonucleotides at the doses required for body-wide therapeutic applications poses a practical and economic challenge.

#### 8.3.3 Self-restricting systems of Cas9

Many self-restricting (also called self-inactivating or self-limiting) systems of CRISPR-Cas9 have been described (189–194). While these systems may differ in their exact mechanisms, the overarching idea centers on delivering an sgRNA that targets the Cas9 vector genome to prevent further Cas9 expression (Figure 3E). The self-restricting strategy was initially developed to reduce off-target editing through the transient expression of Cas9. This strategy should also, in theory, mitigate host immune responses to Cas9. However, there are two main concerns with this strategy. One is reduced on-target editing. Cas9 may begin to inactivate its own genome upon co-expression of Cas9 and gRNA. This immediate decrease in Cas9 expression may compromise the efficiency of target editing. Indeed, Li et al. reported a significant reduction of on-target editing at the Mttp locus when using the self-restricting system (191). Surprisingly, this reduction was not observed at the Ldlr and ApoE loci in the same study, suggesting that other factors may shape on-target editing in self-inactivating systems. The other concern is incomplete abrogation of Cas9 expression. Self-inactivating systems were designed to control the duration of Cas9 expression. However, they may not fully eliminate Cas9 expression in transduced tissues. Incomplete abrogation of Cas9 expression may lead to residual antigen persistence, which could be sufficient to elicit host immune responses toward Cas9. Li et al. detected residual Cas9 expression in the liver up to 6 weeks after administration of self-inactivating AAV Cas9 vectors, suggesting self-inactivating cannot eliminate Cas9 antigen persistence (191). Low levels of residual Cas9 antigen may be sufficient to sustain antigen presentation and restimulate host immune responses, particularly in the context of pre-existing immunity or inflammatory micromilieu.

#### 8.3.4 Transgene silencing

Transgene silencing is a unique and relatively understudied strategy for achieving transient Cas9 expression. It refers to the progressive loss or downregulation of transgene expression over time, despite the continued presence of DNA. Transgene silencing can occur through transcriptional or post-transcriptional mechanisms. Transcriptional silencing has been observed or inferred in multiple AAV studies and CRISPR studies, often mediated through epigenetic regulation of the transgene cassette (195–198). One key regulatory target is the promoter, which drives transgene expression. Consequently, silencing the promoter will abrogate downstream protein expression. Certain viral promoters, such as the cytomegalovirus (CMV) promoter, are particularly susceptible to epigenetic modifications, including DNA methylation, which results in eventual silencing (199–203) (Figure 3F). Consistent with this, Zhu et al. reported that AAV-SaCas9 driven by a CMV promoter exhibited peak mRNA expression at 4 weeks, followed by a decrease at 8 weeks and loss of detectable expression by 12 weeks in murine ear pinnae (197). These observations suggest that the rational engineering of the promoter represents a promising approach to induce transgene silencing for transient Cas9 expression.

In addition to promoter engineering, emerging evidence indicates that the AAV capsid itself plays a role in transcriptional regulation and epigenetic modification of its own genome (195, 196, 204, 205). For example, replacing the VP1 N-terminal domain of avian AAV with that of AAV8 confers transduction in human cells (196). This effect is attributed to the ability of the AAV8 VP1 N-terminal domain to recruit essential host factors to the AAV genome, thereby increasing transcription, chromatin accessibility, and histone methylation. These findings suggest that the role of the capsid helps determine transduction efficiency and helps regulate the epigenetic state of AAV genomes. As such, capsid engineering provides a means to control transgene expression and, in the context of CRISPR-Cas9, modulate antigen persistence and immunogenicity.

### 8.4 Immune modulation

**FIGURE 4:**
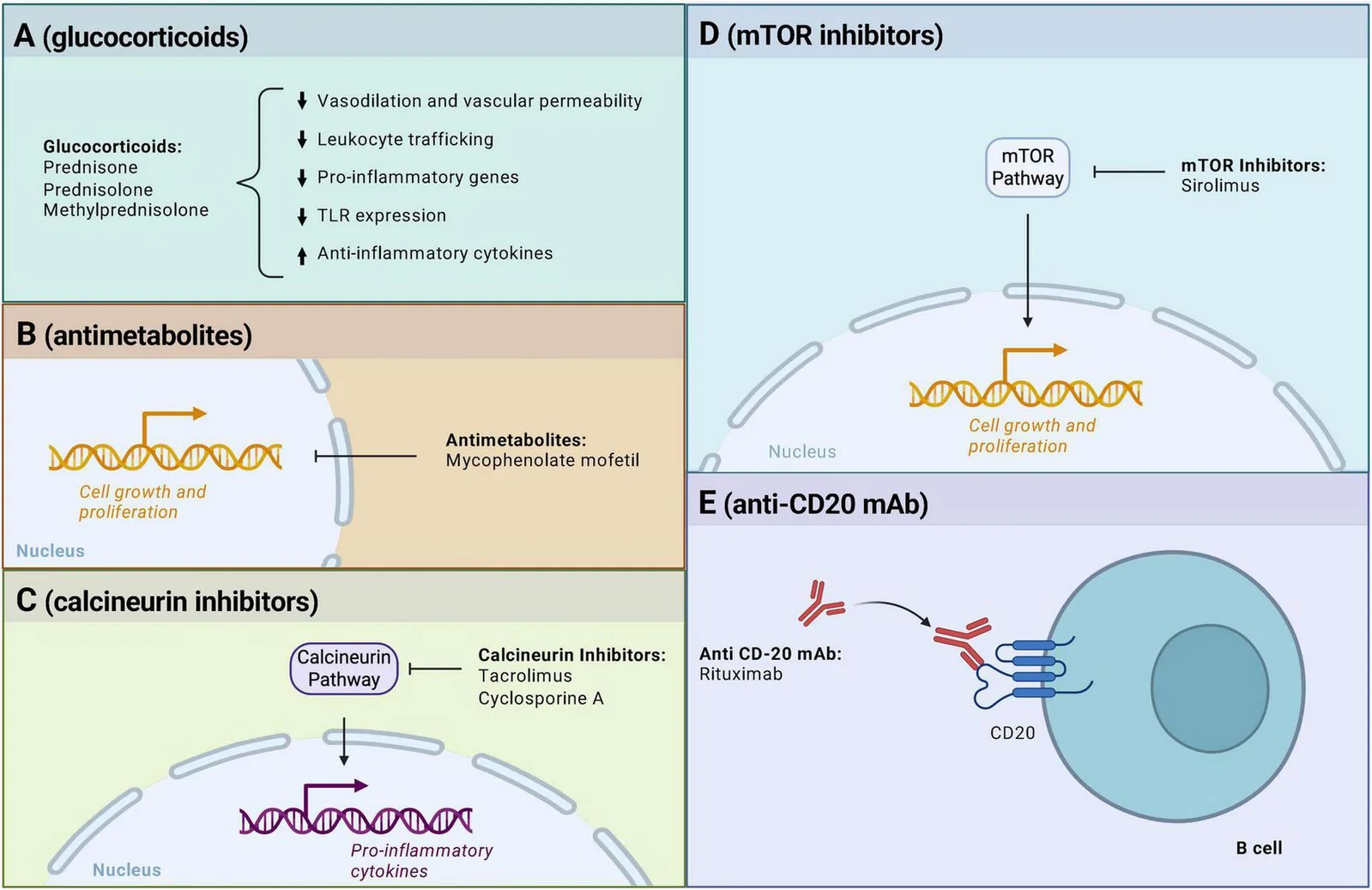
Pharmacological immunosuppression to prevent and suppress host immune responses to Cas9. **(A)** Glucocorticoids. Glucocorticoids, such as prednisone, prednisolone, and methylprednisolone, exert broad anti-inflammatory effects through multiple mechanisms. **(B)** Antimetabolites. Antimetabolites, such as mycophenolate mofetil, act directly within the nucleus to inhibit DNA synthesis, thereby halting cell growth and proliferation. **(C)** Calcineurin inhibitors. T cell receptor activation triggers an influx of extracellular calcium. Elevated cytoplasmic calcium concentration activates the calcineurin pathway and induces proinflammatory cytokine expression. Calcineurin inhibitors, such as tacrolimus and cyclosporine A, can block this pathway. **(D)** mTOR inhibitors. mTOR is a master regulator of cell growth and metabolism. mTOR pathway activation promotes lymphocyte proliferation, differentiation, and survival. **(E)** Anti-CD20 monoclonal antibodies. Anti-CD20 monoclonal antibodies, such as rituximab, target CD20 on the surface of B cells and flag them for destruction.

**FIGURE 5:**
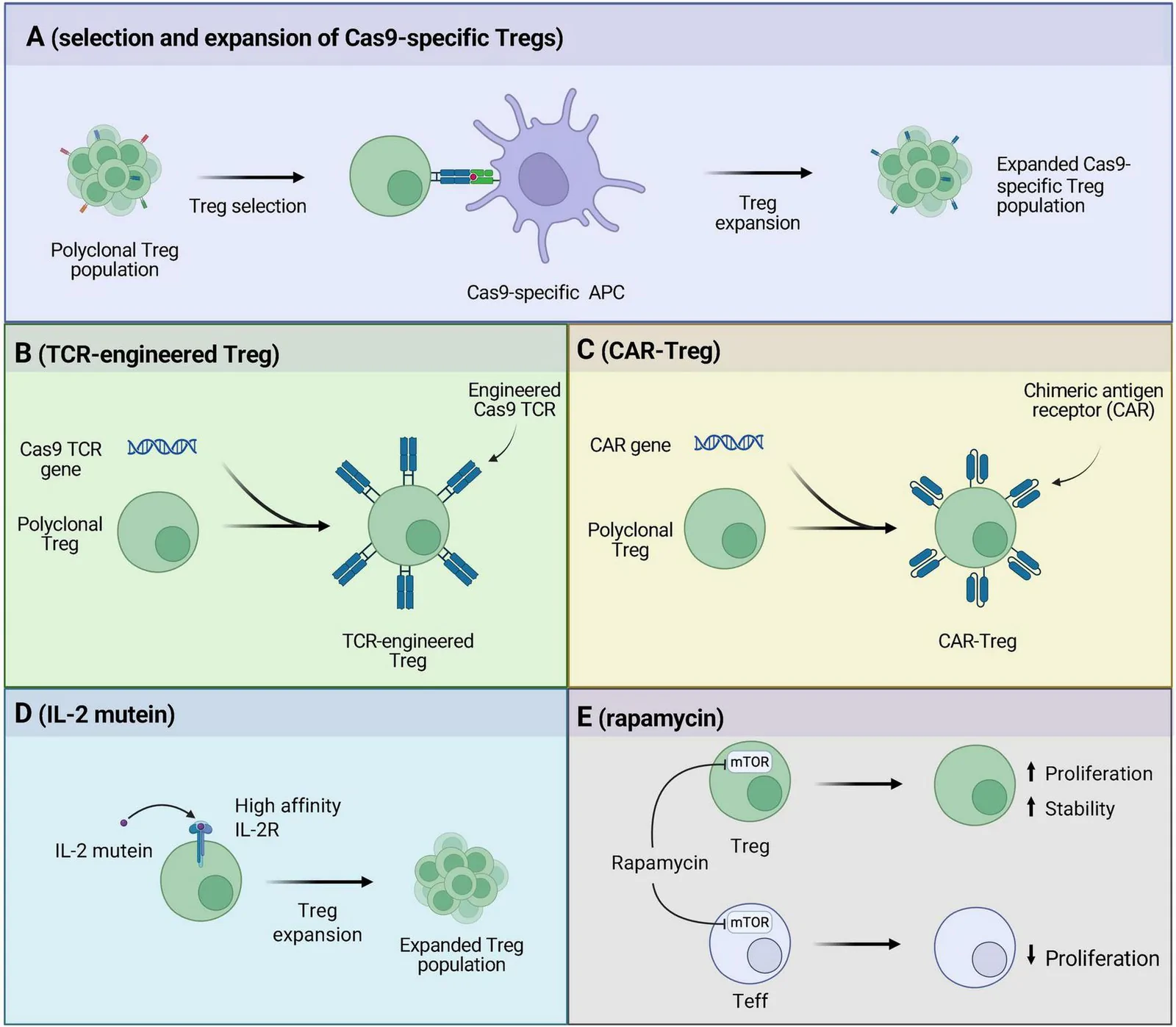
Modulating host immune responses to Cas9 with regulatory T-cells (Tregs). **(A)** Cas9-specific Tregs are expanded from a polyclonal Treg pool by stimulation with Cas9-presenting APCs. **(B)** Polyclonal Tregs are engineered to express a Cas9-specific T cell receptor (TCR). **(C)** Polyclonal Tregs are engineered to express a Cas9-specific chimeric antigen receptor (CAR). **(D)** Treg-selective IL-2 mutein preferentially binds to the high-affinity IL-2 receptor and promotes Treg expansion. **(E)** Rapamycin favors Treg expansion while suppresses effector T cell (Teff) responses.

**FIGURE 6:**
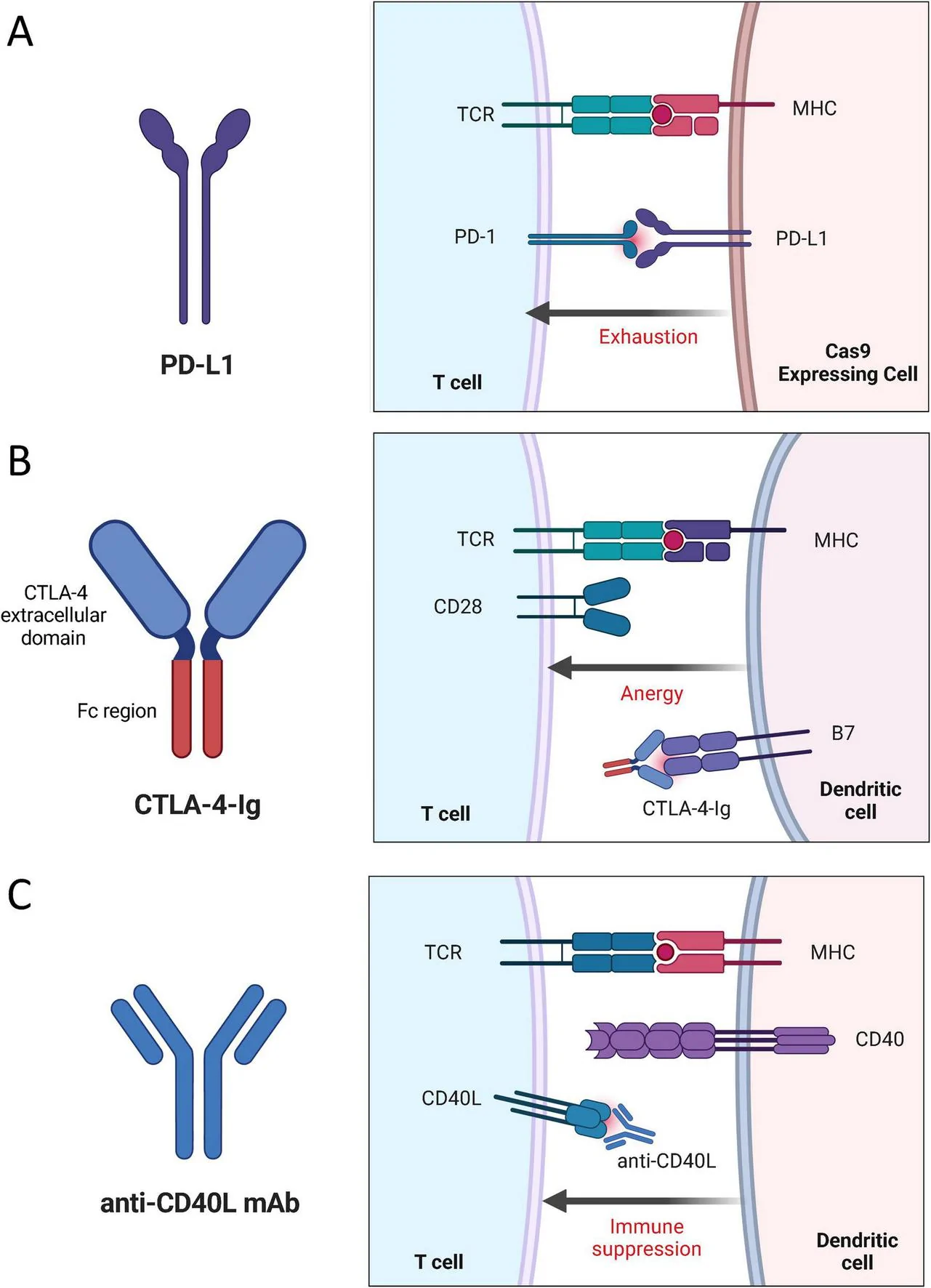
Induction of immunotolerance to Cas9. **(A)** Immune tolerance to Cas9 can be induced by expressing programmed cell death ligand 1 (PD-L1) on the surface of Cas9-expressing cells. PD-L1 promotes T cell exhaustion by engaging programmed cell death protein 1 (PD-1) on interacting T cells. **(B)** Delivery of CTLA-4-Ig fusion protein enables competitive binding of B7 receptors on antigen presenting cells (such as dendritic cells), preventing CD28 co-stimulation, thereby inducing T cell anergy. **(C)** Anti-CD40L monoclonal antibodies can competitively inhibit CD40/CD40L interactions, suppressing downstream immune activation.

The immune response to Cas9 can potentially be mitigated through immune modulation. Immune modulation or immunomodulation refers to the intentional regulation, stimulation, or suppression of the body’s immune system through drugs, biologics, or cell-based therapies (Figures 4–6).

#### 8.4.1 Transient immunosuppression

Suppression of the host immune system and its capacity to combat pathogens may be deliberately induced through pharmacological drugs (Figure 4). Pharmacological immunosuppression is a conventional strategy widely adopted as standard practice in AAV gene therapies to limit innate and adaptive immune responses to the viral capsid and transgene product (206, 207). Corticosteroids such as prednisone, prednisolone, and methylprednisolone are the most used immunosuppressive regimens to inhibit responses to AAV (Figure 4A). Corticosteroids exert broad anti-inflammatory effects through multiple mechanisms, including inhibition of vasodilation and vascular permeability, alteration of leukocyte trafficking, suppression of pro-inflammatory genes, upregulation of anti-inflammatory cytokines, and downregulation of TLR expression (208–211). However, it has been shown that despite an aggressive corticosteroid regimen, dogs administered with AAV-CRISPR mounted a Cas9-specific CTL response (35). Beyond corticosteroids, several other immunosuppressants have been used in AAV gene therapies, including mycophenolate mofetil (MMF), calcineurin inhibitors, sirolimus, and rituximab. MMF is a prodrug of mycophenolic acid (MPA) (Figure 4B), which inhibits inosine-5′-monophosphate dehydrogenase (IMPDH). IMPDH is the rate-limiting enzyme for *de novo* guanosine nucleotide synthesis, a highly important pathway for T and B lymphocyte proliferation (212). Thus, MMF suppresses cell-mediated and humoral immune responses through inhibition of T and B cell proliferation. Calcineurin inhibitors (Figure 4C), most notably tacrolimus and cyclosporine A, bind their respective intracellular immunophilins (FKBP12 and cyclophilin) to block calcineurin from dephosphorylating nuclear translocation of nuclear factor of activated T cells (NFAT). This leads to the subsequent suppression of proinflammatory cytokines such as IL-2, which effectively suppresses T cell proliferation and T helper cell-dependent B cell responses (213–215). Like tacrolimus, sirolimus (rapamycin) also binds to FKBP12. However, the resulting complex inhibits the mechanistic target of rapamycin (mTOR), a central kinase involved in lymphocyte proliferation and differentiation (214) (Figure 4D). Inhibition of mTOR may lead to broad suppression of T and B cell responses. Lastly, rituximab is an anti-CD20 monoclonal antibody that targets and destroys B cells (216) (Figure 4E). It has been explored as a strategy to reduce pre-existing neutralizing antibodies against AAV capsids. However, this strategy has only demonstrated partial efficacy and should be used in conjunction with other immunosuppressive drugs (217). A recent approach combining anti-CD20 with anti-B-cell activating factor (BAFF) appears promising for the prevention of antibody formation against AAV and may, if timed properly, lower the risk of T cell activation against the transgene product (218). Overall, immunosuppression alone may not contain adaptive immune responses toward Cas9. New combinatorial regimens and compounds should be investigated.

#### 8.4.2 Regulatory T-cell therapy

Regulatory T cell (Treg) therapies are emerging as promising therapeutics for treating autoimmune and inflammatory diseases (219–223). The ongoing success of Treg therapies represents a compelling argument for their use in CRISPR-based gene therapy to establish antigen-specific tolerance to Cas9. Tregs are a specialized subset of CD4+ T lymphocytes that maintain immune homeostasis by regulating excessive immune responses. They are characterized by the surface display of CD25 and the expression of master transcription factor FOXP3 (224). They mediate suppression through multiple mechanisms, including the secretion of anti-inflammatory cytokines such as IL-10, IL-35, and TGF-beta, as well as cytotoxic T-lymphocyte-associated protein 4 (CTLA-4)-mediated downregulation of costimulatory molecules on APCs (225, 226). Given their pivotal role in maintaining peripheral tolerance, harnessing Treg populations through selective expansion or genetic engineering represents a biologically grounded strategy for attenuating adaptive immune responses against Cas9 (15) (Figure 5).

The most direct approach is adoptive Treg transfer. Wagner et al. found that a significant fraction (11%–71%) of SpCas9-reactive CD4+ CD137+ T cells expressed Treg markers in human PBMCs (15). These Cas9-reactive Tregs can be isolated and expanded *ex vivo* using Cas9 peptide-loaded APCs and then reinfused (220) (Figure 5A). This can be performed using autologous Tregs derived from the patient or allogeneic Tregs harvested from a donor. Autologous transfer minimizes the risk of rejection and graft-versus-host complications. Allogeneic Tregs offer advantages in scalability and off-the-shelf availability but may require concurrent immunosuppression to ensure Treg persistence. A key challenge for both methods is maintaining FOXP3 expression and suppressive function following expansion, as Tregs may lose lineage stability under pro-inflammatory conditions (227, 228).

A more targeted approach involves the engineering of harvested autologous Tregs to express either an engineered TCR specific for Cas9 epitopes or a chimeric antigen receptor (CAR) for Cas9-expressing cells (223, 229) (Figures 5B, C). This strategy is conceptually analogous to approaches explored in AAV gene therapy, where CAR-Tregs are being investigated to prevent and modulate immune responses toward AAV capsid and its transgene (230). By specifically directing Treg activity against the Cas9 antigen or specific surface proteins of Cas9-expressing cells, engineered TCR-Treg or CAR-Treg may achieve more precise and durable suppression than polyclonal Treg. A major downside of this approach is its manufacturing complexity and the cost associated with TCR and CAR engineering.

A less resource-intensive and readily integrated approach is the *in vivo* expansion of endogenous Treg populations using engineered IL-2 variants (231–233) (Figure 5D). Conventional IL-2 signals through both the high-affinity IL-2 receptor that is constitutively expressed on Tregs and the intermediate-affinity IL-2 receptor that is highly expressed on conventional T cells and NK cells, limiting its therapeutic window (233). Mutein IL-2 variants are engineered to selectively bind the high-affinity IL-2 receptor, thereby preferentially expanding Treg populations while minimizing stimulation of conventional T cells and NK cells. This selectivity makes mutein IL-2 a relatively accessible strategy that can be readily combined with other immunomodulatory approaches without the complexity of *ex vivo* cell engineering (234, 235).

Rapamycin further complements T-reg based strategies through a dual mechanism (236, 237) (Figure 5E). Rapamycin inhibits the mTOR pathway downstream of IL-2/PI3K signaling in both CD4+ and CD8+ T cells, thereby reducing effector T cell proliferation. Rapamycin also promotes Treg expansion and stability by upregulating FOXP3 expression in naïve CD4+ T cells in the presence of IL-2 and TGF-beta (238–241). Importantly, the preferential effect of rapamycin is IL-2 dependent, as abolishing IL-2 signaling may negate Treg expansion. This mechanistic complementarity makes the combination of rapamycin and mutein IL-2 particularly powerful, as the mutein IL-2 provides a Treg-selective proliferative signal, while rapamycin suppresses effector T cell proliferation and increases FOXP3 expression, thereby promoting Treg differentiation.

#### 8.4.3 Induction of immunotolerance

Immunotolerance is a state of unresponsiveness to a specific antigen, maintained through either central or peripheral mechanisms (242–244). Unlike broad immunosuppression, which non-selectively dampens immune activity, true immunotolerance reprograms the immune system to ignore defined antigens without compromising global immune competence. Several strategies may be employed to induce immunotolerance to Cas9 (Figure 6).

One approach is the co-expression of immunoinhibitory molecules in Cas9-expressing cells (Figure 6A). Expression of PD-L1 is a candidate strategy for reducing immune-mediated clearance of transduced cells (245). PD-L1 is the ligand of programmed cell death protein 1 (PD-1), an inhibitory receptor expressed on T cells that leads to the suppression of immune signaling and promotes T cell exhaustion upon binding. PD-L1 overexpression on the surface of target cells to exhaust effector T cells is a promising immune evasion strategy that has been well characterized in the context of tumor immune evasion (246). Co-expression of PD-L1 with Cas9 from the same transgene expression cassette can ensure concurrent display of PD-L1 with Cas9 peptide-loaded MHC. This expression of PD-L1 with antigenic pMHC-I sends a mixed signal. Engagement of PD-L1 with PD-1 recruits phosphatases such as SHP-2 to shut down activating signals in CD8+ T cells, leaving the cell in a paralyzed state of exhaustion (246, 247).

Peripheral immune tolerance to Cas9 may be achieved through hepatic tolerance induction that may be carried out through persistent expression of Cas9 in the liver. Persistent antigen exposure within the liver promotes peripheral tolerance and supports local expansion of Tregs (109, 248, 249). These Tregs will actively suppress effector T cells from attacking target tissues that display the antigen. However, successful tolerance induction requires meeting several criteria, including avoiding expression in professional APCs, maintaining minimal transgene expression, and minimizing innate immune responses (248).

A further approach involves the exogenous delivery or vector-mediated expression of costimulation blockers such as CTLA-4-Ig and anti-CD40L (Figures 6B, C). These immunomodulatory molecules operate by inhibiting the “second signal” required for T-cell activation. In the absence of costimulation, antigen recognition through the TCR alone is insufficient to drive productive T cell priming and instead favors T cell anergy. For example, CTLA4-Ig is a soluble fusion protein containing the extracellular domain of CTLA-4 and an IgG Fc region (Figure 6B). Through high-affinity binding to the B7 molecules (CD80 and CD86) on APCs, it blocks CD28-dependent costimulation and attenuates T cell activation (250, 251). Loss of CD28 costimulatory signaling in T cells will thereby prevent full T cell activation (252). A recent preclinical study supports the potential of CTLA4-Ig to limit immune responses against the gene transfer vector and transgene product (253). On the other hand, anti-CD40L is a monoclonal antibody that blocks the interaction between CD40L on activated CD4^+^ T helper cells and CD40 on APCs (Figure 6C). By disrupting this critical costimulatory pathway, anti-CD40L inhibits APC activation and downstream T-cell priming, thereby limiting sustained cytotoxic T-cell responses (254, 255). Together, these two blockers act on complementary sides of the costimulatory axis, and their combination has been shown to induce tolerance in hepatic gene therapy studies (248).

## 9 Conclusions and future perspectives

Cas9 immunogenicity remains a major obstacle to the successful clinical translation of CRISPR-based therapies. As these therapies advance into clinical settings, immune responses to Cas9 have emerged not merely as a theoretical concern, but a real-world challenge with significant implications for safety and efficacy. Given the diversity of clinical contexts, no single immunological framework can fully recapitulate the range of Cas9-directed immune responses. Moreover, Cas9 immunogenicity is governed by a complex interplay among antigen persistence, antigen load, delivery modality, route of administration, tissue microenvironment, and host immune background and status. The context-dependent interactions of these key determinants remain incompletely understood.

Several conclusions can be drawn from the current body of evidence on Cas9 immunity. First, in the *ex vivo* autologous setting (such as CASGEVY), Cas9 immunogenicity can be effectively avoided since the antigen is absent in the final therapeutic product. However, this is not the case for *in vivo* Cas9 editing therapy. Second, delivery to immune-privileged sites, such as the subretinal space, can substantially reduce Cas9-specific responses, as demonstrated by EDIT-101. However, this protection appears to be incomplete, and its generalizability to other tissues, Cas9 orthologs, and higher antigen loads remains uncertain. Third, LNP-mediated hepatic delivery of Cas9 mRNA leverages transient Cas9 expression to achieve robust editing with a generally manageable safety profile, as shown in NTLA-2001 and NTLA-2002. However, the hepatic adverse event reported in the MAGNITUDE phase 3 trial underscores that even with transient expression, multiple factors such as patient age, underlying disease state, and potential contributions from Cas9-specific cellular immunity may lead to immune-mediated toxicities in unanticipated ways (67). Together, these findings highlight the need for continued, rigorous clinical evaluation before such approaches can be considered broadly safe.

The mitigation strategies discussed here collectively tackle Cas9 immunogenicity from multiple mechanistic angles, including reducing the intrinsic immunogenicity of the Cas9 protein, restricting its expression to the intended target cells, minimizing innate immune sensing against its payload, limiting the duration of antigen exposure, and modulating the host immune responses at the levels of innate sensing, adaptive priming, and effector function. While each strategy appears promising, none is likely to be sufficient in isolation. For example, immunosilenced Cas9 may reduce T cell recognition of immunodominant epitopes; however, given the breadth of HLA-restricted epitope presentation across diverse patient populations make it unlikely to fully eliminate immunogenicity by modification of a limited number of residues. Self-restricting or inducible expression systems offer temporal control over Cas9 expression, but leaky expression or lingering expression due to incomplete abrogation may limit their clinical applicability. Immunomodulatory strategies are mechanistically compelling, yet the durability of induced tolerance and their translation to clinical settings, especially in the context of high antigen load or pre-existing immunity, has yet to be established. Collectively, these considerations argue for a shift from single-strategy evaluation to systematic investigation of combinatorial approaches. For instance, combining an immunosilenced Cas9 variant with miR-142-3p mediated APC-specific transcript suppression, tissue-specific promoter control, and a self-restricting expression system, alongside a short course of rapamycin and IL-2 mutein therapy, is mechanistically coherent. Each component addresses a distinct step of the immune activation cascade, yet such combinations have not been rigorously tested in clinically relevant models.

Realizing the potential of combinatorial approaches will require continued innovation across several fronts. Advances in next-generation CRISPR systems, including high-fidelity Cas9 variants, may achieve sufficient levels of on-target gene editing at lower doses, potentially reducing antigen load and associated immunogenic risk. Furthermore, emerging Cas orthologs with distinct immunogenic profiles, such as Cas12a, Cas13, and Casφ, may broaden the CRISPR therapy toolkit and enable ortholog selection as an immune-mitigation strategy. AI-assisted immune epitope prediction and protein engineering represent a particularly promising approach. The generation of novel immunosilenced Cas9 variants tailored to specific HLA haplotypes may further advance the prospects of personalized CRISPR therapies. Advances in protein engineering more broadly may yield functional Cas9 nucleases with enhanced immune evasion properties. Development of next-generation delivery technologies may also favorably control several key determinants of Cas9 immunogenicity. For example, employing myotropic AAV capsids for CRISPR DMD therapy may enable lower vector doses to be delivered while achieving relevant levels of dystrophin restoration and improved skeletal muscle targeting (256).

Achieving long-lasting CRISPR-based therapies will require not only advances in CRISPR system engineering and immune mitigation strategies but also the development of preclinical models that adequately reflect the immunological complexity of human patients, enabling more reliable evaluation of these approaches. An equally important issue is the development of validated and standardized immunological monitoring frameworks for preclinical research and clinical trials. These should encompass pre-treatment HLA haplotyping, screening for pre-existing Cas9-specific T cells, anti-Cas9 and anti-vector antibody profiling, longitudinal measurement of cellular immune responses, and histological assessment of edited tissues. The lack of standardized frameworks has made cross-trial comparisons difficult and limited the field’s ability to build upon mechanistic understanding from both preclinical and clinical data. Establishing a consensus framework for immunological monitoring would improve cross-study comparability and substantially advance understanding of Cas9-directed immune responses and their impact on patient outcomes.

Ultimately, the development of safe and efficacious CRISPR-based therapies will require immune mitigation to be integrated into therapeutic design rather than treated as an afterthought. This necessitates deliberate consideration, at the earliest stage of therapeutic development, of disease context, vector and delivery modality, Cas9 engineering, promoter and regulatory element design, dosing strategy, route of administration, and the use of adjunct immunosuppressive regimens or adjunct cell therapies. Continued interdisciplinary innovation spanning genome engineering, delivery systems, and immunological safety will be imperative for the successful clinical translation of CRISPR therapies across a broad range of diseases.
